# Transmembrane Prolyl 4-Hydroxylase is a Novel Regulator of Calcium Signaling in Astrocytes

**DOI:** 10.1523/ENEURO.0253-20.2020

**Published:** 2021-01-05

**Authors:** Nadiya Byts, Subodh Sharma, Jenny Laurila, Prodeep Paudel, Ilkka Miinalainen, Veli-Pekka Ronkainen, Reetta Hinttala, Kid Törnquist, Peppi Koivunen, Johanna Myllyharju

**Affiliations:** 1 Oulu Center for Cell-Matrix Research, Biocenter Oulu and Faculty of Biochemistry and Molecular Medicine, University of Oulu, Oulu 90014, Finland; 2 Biocenter Oulu, University of Oulu, Oulu 90014, Finland; 3 PEDEGO Research Unit, Faculty of Medicine, University of Oulu, Oulu 90014, Finland; 4 Cell Biology, Faculty of Science and Engineering, Åbo Akademi University, Turku 20500, Finland; 5 Minerva Foundation Institute for Medical Research, Biomedicum Helsinki, Helsinki 00290, Finland

**Keywords:** calcium signaling, hypoxia-inducible factor, prolyl 4-hydroxylase, vesicular exocytosis

## Abstract

Prolyl 4-hydroxylases (P4Hs) have vital roles in regulating collagen synthesis and hypoxia response. A transmembrane P4H (P4H-TM) is a recently identified member of the family. Biallelic loss of function P4H-TM mutations cause a severe autosomal recessive intellectual disability syndrome in humans, but functions of P4H-TM are essentially unknown at cellular level. Our microarray data on *P4h-tm*^−/−^ mouse cortexes where P4H-TM is abundantly expressed indicated expression changes in genes involved in calcium signaling and expression of several calcium sequestering ATPases was upregulated in *P4h-tm*^−/−^ primary mouse astrocytes. Cytosolic and intraorganellar calcium imaging of *P4h-tm*^−/−^ cells revealed that receptor-operated calcium entry (ROCE) and store-operated calcium entry (SOCE) and calcium re-uptake by mitochondria were compromised. HIF1, but not HIF2, was found to be a key mediator of the P4H-TM effect on calcium signaling. Furthermore, total internal reflection fluorescence (TIRF) imaging showed that calcium agonist-induced gliotransmission was attenuated in *P4h-tm*^−/−^ astrocytes. This phenotype was accompanied by redistribution of mitochondria from distal processes to central parts of the cell body and decreased intracellular ATP content. Our data show that P4H-TM is a novel regulator of calcium dynamics and gliotransmission.

## Significance Statement

Transmembrane prolyl 4-hydroxylase (P4H-TM) is a recently identified member of the P4H family. Biallelic loss of function P4H-TM mutations cause a syndromic form of severe cognitive impairment in humans. Here, we demonstrate for the first time that inactivation of P4H-TM in astrocytes disturbs calcium signaling in a HIF1-dependent manner. The observed changes in calcium signaling were accompanied by attenuated vesicular exocytosis. These findings suggest that abnormal calcium signaling resulting from P4H-TM inactivation may be involved in the molecular basis of a severe human intellectual disability syndrome. Therefore, future studies to unravel the exact molecular mechanisms how P4H-TM affects calcium signaling and what effects P4H-TM has on astrocyte functions in healthy and various disease conditions will be of interest.

## Introduction

The prolyl 4-hydroxylase (P4H) family of enzymes includes the collagen P4Hs and hypoxia-inducible factor (HIF) P4Hs that have vital roles in collagen synthesis and in the regulation of the hypoxia response, respectively (Myllyharju, 2008, 2013; Ratcliffe, 2013; Ivan and Kaelin, 2017). HIFs (HIF1, HIF2, and HIF3) are heterodimeric transcription factors consisting of an oxygen-sensitive α-subunit and a constitutive β-subunit. HIF-P4Hs hydroxylate two prolyl residues located in the oxygen-dependent degradation domain (ODDD) of the HIFα subunit in normoxia. Hydroxylation leads to von Hippel–Lindau (VHL)-targeted degradation of HIFα, which suppresses the transcription of hypoxia responsive genes in normoxia. In contrast, HIF-P4Hs are inactivated in hypoxia, which leads to stabilization and accumulation of HIF and induction of hypoxia responsive genes. Originally three members of the HIF-P4H family were identified: HIF-P4H-1, HIF-P4H-2, and HIF-P4H-3 (also known as PHD1, PHD2, and PHD3 or EGLN2, EGLN1, and EGLN3; Myllyharju, 2013; Ratcliffe, 2013; Ivan and Kaelin, 2017).

Our laboratory was among the first to clone and characterize a human transmembrane P4H (P4H-TM), a distinct member of the P4H family (Oehme et al., 2002; Koivunen et al., 2007). P4H-TM contains a transmembrane domain toward its N terminus and we showed that it is located in the endoplasmic reticulum (ER) membranes with its catalytic site inside the lumen (Koivunen et al., 2007). The cellular location suggested that P4H-TM could be a collagen P4H and the P4H-TM amino acid sequence also resembled more closely those of the collagen P4Hs than the HIF-P4Hs (Koivunen et al., 2007). However, P4H-TM lacked the peptide-substrate-binding domain of the collagen P4Hs (Myllyharju and Kivirikko, 1999), and it did not hydroxylate collagen polypeptides *in vitro* (Koivunen et al., 2007). Instead, like HIF-P4Hs, P4H-TM has been shown to regulate the oxygen-dependent stability of HIFα *in cellulo* and to hydroxylate the HIFα ODDD *in vitro* (Koivunen et al., 2007). However, P4H-TM did not show as strict target proline specificity in the hydroxylation of the HIFα ODDD as the HIF-P4Hs (Koivunen et al., 2007). Analyses of *P4h-tm*^−/−^ mice have shown that P4H-TM affects erythropoiesis (Laitala et al., 2012), tumor angiogenesis (Klotzsche-von Ameln et al., 2013), renal and retinal development (Leinonen et al., 2016) and behavior (Leinonen et al., 2019) in mouse, and that some of these phenotypic abnormalities are not likely to be HIF-mediated. Therefore, it has been suggested that P4H-TM may have additional, yet uncharacterized substrates (Koivunen et al., 2007; Leinonen et al., 2016, 2019).

P4H-TM was shown to be highly expressed in the brain in comparison to other tissues (Koivunen et al., 2007; Leinonen et al., 2016), nevertheless its function in the brain is unknown. The cellular functions of P4H-TM in brain cells are of considerable interest because loss-of-function P4H-TM mutations have been shown to cause a human HIDEA syndrome characterized by hypotonia, intellectual disability, and eye abnormalities (Kaasinen et al., 2014; Rahikkala et al., 2019). Therefore, in the current study we sought to reveal cellular functions and pathways controlled by P4H-TM in brain cells, which potentially could affect brain function.

Taking into consideration the important secretory role of astrocytes in the brain and their ability to signal to neurons and neighbor astrocytes through the vesicular release of neuroactive and glia active substances (gliotransmission) in a calcium-dependent manner (Bezzi and Volterra, 2001; Oliet et al., 2001; Lalo et al., 2014; Vardjan and Zorec, 2015), we chose astrocytes as a cellular system. We investigated the effects of the lack of P4H-TM on calcium dynamics and vesicular exocytosis by imaging live cortical astrocytes from wild-type (WT) and P4H-TM knock-out (KO) mice and demonstrated the importance of P4H-TM for both processes. Our data also show that HIF1 is the key mediator of this P4H-TM function.

## Materials and Methods

### Animals and ethics approval

*P4h-tm*^−/−^, *Hif-p4h-1*^−/−^, *Hif-p4h-3*^−/−^, and *Hif-p4h-2* hypomorph mice (Hyvärinen et al., 2010; Laitala et al., 2012; Ullah et al., 2017) and their corresponding WT controls were used for cortical dissection and isolation of primary astrocytes. In the hypomorph *Hif-p4h-2* mouse line (full KO of this gene is embryonic lethal) the *Hif-p4h*-2 gene is disrupted by a GeneTrap (gt) insertion cassette, but because of partial skipping of the insertion cassette varying amounts of WT *Hif-p4h-2* mRNA is generated from the gene-trapped alleles in different tissues (Hyvärinen et al., 2010). Animal experiments were approved by the Animal Experiment Board of Finland, following the regulations of the EU Directive 86/609/EEC, the European Convention ETS123, and the national legislation of Finland. The recommendations given by the Federation of European Laboratory Animal Science Associations and the Finnish and EU legislations concerning laboratory animal experiments and handling were followed.

### Microarray

The GeneChip experimental procedures were performed according to the Affymetrix GeneChip Expression Analysis Technical Manual. Shortly, total RNA was extracted from cortical tissue using TriPure isolation reagent (Roche Applied Science). Double-stranded DNA was synthesized using 8 μg of total RNA as a template by means of the One-cycle cDNA synthesis kit (Affymetrix) and T7-(dT)24 primer, and the DNA was purified using the GeneChip Sample Cleanup Module (QIAGEN). *In vitro* transcription was performed to produce biotin-labeled cRNA using an IVT labeling kit (Affymetrix) according to the manufacturer’s instructions. Biotinylated cRNA was cleaned with a GeneChip Sample Cleanup Module (QIAGEN), fragmented to 35–200 nt, and hybridized to Affymetrix Mouse Genome 430_ 2.0 arrays, which contain ∼45,000 mouse transcripts. After washing, the array was stained with streptavidin–phycoerythrin (Invitrogen), and the staining signal was amplified with biotinylated anti-streptavidin (Vector Laboratories) and a second staining with streptavidin–phycoerythrin and then scanned on a GeneChip Scanner 3000. Hybridization signal intensities were quantified using Affymetrix GeneChip Operating System (Affymetrix). CEL files and the probe annotation files were downloaded, and the gene expression data of all samples were normalized using the GenePattern software (freely available software package developed at the Broad Institute of MIT and Harvard (http://genepattern.broadinstitute.org; Reich et al., 2006). Normalized expression ratio data were further analyzed using the Gene Set Enrichment Analysis (GSEA) to identify significantly enriched groups of genes. Kyoto Encyclopedia of Genes and Genomes (KEGG) database and Reactome Knowledgebase was used for analysis and expression values between WT and P4H-TM KO cortexes were compared. Gene datasets were considered to be significantly enriched according to GSEA default settings, *p *<* *0.05.

### Data availability

The microarray data have been deposited in the NCBI Gene Expression Omnibus (GEO; Edgar et al., 2002) and are accessible through GEO Series accession number GSE126425.

### Primary cortical astrocytes

Primary cortical astrocyte cultures were prepared as described (McCarthy and de Vellis, 1980) from 1- to 2-d-old *P4h-tm*^−/−^, *Hif-p4h-1*^−/−^, *Hif-p4h-2* hypomorph, and *Hif-p4h-3*^−/−^ mice and their WT controls. The pups were taken for cell culture isolation regardless of their gender. The mice were killed via decapitation, and cortexes were removed of the meninges, dissected and trypsinized. After mechanical trituration, the cell suspension was passed through a 40-μm cell strainer and plated on poly-d-lysine-coated dishes in a density of 25,000 cells/cm^2^ in DMEM (Lonza) containing 1 g/l glucose and supplemented with 1% penicillin/streptomycin and 20% fetal calf serum (FCS). Cultures were established from cells pooled from one to four animals of the same genotype. The cultures were grown at 37°C under 5% CO_2_/95% air and 90% humidity in 10% FCS-containing medium, with medium being exchanged every second day. After 9–10 d, the cells were trypsinized and passaged. The cells were used from the first or second passage (two to three weeks in culture) for the experiments. Enrichment for astroglial cells was ∼90% under these culture conditions, microglia content was ∼4% and neuronal content ∼2% as detected by immunocytochemistry for GFAP, CD11b, and β-Tubulin III cell-type markers, respectively (data not shown). The cells were treated with ATP (Sigma), thapsigargin (TG; BioVision), 2-aminoethoxydiphenyl borate (2-APB; Tocris), ionomycin (BioVision), puromycin (Sigma), anisomycin (Sigma), and EGTA (Sigma). The treatment times and doses used are described in the figure legends of the respective experiments.

### Oxygen-glucose deprivation (OGD) treatment

In OGD treatment, medium was changed to DMEM containing no glucose (ThermoFisher) and supplemented with 1% penicillin/streptomycin and 10% FCS and the cells were grown at 37°C under 5% CO_2_/1% O_2_ for the periods of time indicated in the figure legends.

### qRT-PCR

Total RNA was isolated using TriPure isolation reagent (Roche Applied Science) and further purified with an EZNA total RNA kit (Omega Biotek), and reverse transcription was performed with an iScript cDNA synthesis kit (Bio-Rad Laboratories). qRT-PCR was performed with iTaq Universal SYBR Green Supermix (Bio-Rad Laboratories) and a CFX96 Touch real-time PCR detection system, using primer sets listed in Table 1.

**Table 1:** Sequences of the qRT-PCR primers

Gene	Forward primer	Reverse primer
P4H-TM	AGCCAGTGCCAACCTTG	AAGCCTGGGATTTCAAAAAG
β-Actin	CAATAGTGATGACCTGGCCGT	AGAGGGAAATCGTGCGTGAC
PMCA2	AGAGATAGACCACGCAGAGC	CTGGGAATCTTCGATCCGGA
PMCA3	GCAGGACGTGACTCTCATCA	CACCAGACACATTCCCACAG
PMCA4	CTTAATGGACCTGCGAAAGC	ATCTGCAGGGTTCCCAGATA
Orai1	TTACTCCGAGGTGATGAGCC	TGGTGGGTAGTCATGGTCTG
Orai2	AGCTACCTGGAACTCGTCAC	CAAACAGATGCACGGCTACC
Orai3	CCAACGACTGCACAGATACG	GCTTTGGAAGGCTGTTGTGA
STIM1	ACGATGCCAATGGTGATGTG	CACCTCATCCACAGTCCAGT
STIM2	CAATCGTGCCACAGTTTCCA	GGCAACTTGACACAGACAGG
SEC61A	GCTCCTGTGCATTCTTCTCC	GGAAGTCAGCCAGGACAGAG
SEC61B	CCGTTCTTAGGCATCAGCAT	TTCTCTGCCGAACAGTGGAT
SEC61G	CGGTTCTCTCCTGAGCTACG	TAACCAGCCGAATTGAGTCC
SERCA2A	GCTCCATCTGCTTGTCCATG	TTTCGGGCCACAAACTTGAG
SERCA2B	CTGTGGAGACCCTTGGTTGT	CAGAGCACAGATGGTGGCTA
SERCA3	CCACTCTCCTGCATCTCCTC	TCCAATCCCTCAGACACACA
SPCA1	Quantitect primer assays (QIAGEN: QT00166278, QT01542072, QT01542079)	
MCU	CGCCAGGAATATGTTTATCCA	CTTGTAATGGGTCTCTCAGTCTCTT
MICU1	GAACTAGCTGTGGGCTCTCG	GGTGGCAAAATATCGGAAAA
MCUR1 HIF1α	AGCCCTCAGAGCAGAAAATG GGCGAGAACGAGAAGAAAAA	CCAGCATCTTCTTCTCGTTCA AAGTGGCAACTGATGAGCAA

### Western blot analysis

To prepare protein samples from primary cortical astrocytes for Western blot analysis, the cells were scraped in lysis buffer (50 mm Tris-HCl, pH 8.0, 50 mm NaCl, 1% Triton X-100, and 1 mm dithiothreitol) supplemented with protease and phosphatase inhibitor cocktails (Roche) at 4°C. The samples were homogenized via mechanical trituration through a 27-G needle, and the lysates were subjected to SDS-PAGE analysis. The protein concentration of the samples was determined using a Bio-Rad protein assay dye reagent concentrate (Bio-Rad Laboratories) or NanoDrop 2000 spectrophotometer (ThermoScientific). For detection of small molecular weight proteins, such as SEC61B and SEC61G subunits, the samples were loaded on 10–20% Mini-Protean Tris-Tricine Gels (Bio-Rad Laboratories), while for detection of higher molecular weight proteins, such as HIF1α, SERCA2 and PMCA3, the samples were loaded on 8% Tris-glycine SDS-PAGE gels. Otherwise, 10% SDS-PAGE gels were used. Proteins were transferred onto nitrocellulose or PVDF membranes using standard methods. The blots were probed with antibodies recognizing ATP2A2/SERCA2 (Cell Signaling, D51B11), ATP2A3/SERCA3 (Boster Biological Technology, RP1055), PMCA2 (ATP2B2, St John’s Laboratory, STJ28955), PMCA3 (G-6; Santa Cruz, sc-390148), SEC61A (Abcam, ab183046), SEC61B (Protein Tech, 15 087-I-AP), SEC61G (Protein Tech, 11147-2-AP), phospho-elF2α pSer51 (Thermo Scientific, MA5-15 133), elF2α (Invitrogen, AHO1182), diphosphorylated Erk1/2 (Sigma-Aldrich, M8159), Erk1/2 (Sigma-Aldrich, M5670), phospho-p38 MAPK (Cell Signaling Technology, 4511S), p38 MAPK (Cell Signaling Technology, 9212S), NDUFS3 (Abcam, ab14711), ATP5A (Abcam, ab14748), UQCRC2 (Abcam, ab14745), COX I (Molecular Probes, A6403), SDHA (Abcam, ab14715), HIF1α (Abcam, ab2185), HIF2α (Abcam, ab199), GFP (Abcam, ab13970), or synaptobrevin 2 (Syb2; Vamp2, Abcam, ab3347). Staining for β-actin (Novus Biologicals, NB600-501) was used as a control for protein loading. The blots were quantified using Fiji-ImageJ software (a Java-based public domain software). The densitometry data were normalized to β-actin.

### Live cell imaging of cellular calcium dynamics

Astrocytes were grown on cell culture dishes with a glass bottom (Greiner; 65,000 cells/cm^2^). The cells were loaded with Fluo-4 A.M. (4 μm, Invitrogen) for 20 min and then incubated for a further 30 min in 37°C before measurement. Imaging was performed in normal extracellular solution (NES) containing 136 mm NaCl, 2.5 mm KCl, 10 mm HEPES, 1.3 mm MgCl_2_, 10 mm glucose, and 2 mm CaCl_2_, pH 7.3 (Royle et al., 2008) as described previously (Terunuma et al., 2015). In some experiments, extracellular calcium was chelated with 2 mm EGTA added into the buffer. Fluorescence images were acquired with a Zeiss Cell Observer Spinning Disk Confocal microscope using epifluorescence illumination (excitation filter bandpass 470/20 nm, emission 525/50 nm), LD LCI Plan-Apochromat 25×/0.8W objective, Zen 2012 Blue software (Carl Zeiss) and Hamamatsu ORCA-R2 camera (Hamamatsu). Images were captured at 1-s intervals for up to 2 min (in some experiments up to 4 min) in 37°C and 5% CO_2_. Image data were analyzed by Zen 2012 Blue software and subsequently by OriginPro 2016 software (OriginLab). The change in intracellular free calcium concentration ([Ca^2+^]_i_) is represented by relative fluorescence intensity [(F_1_ – F_0_)/F_0_, relative unit (r.u.)] (F_0_, at rest; F_1_, after administration of drugs, background subtracted) in the selected cytoplasmic or nuclear parts of the cells.

### Intraorganellar calcium imaging

Astrocytes were grown on cell culture dishes with a glass bottom (Greiner; 65,000 cells/cm^2^). To assess ER Ca^2+^ concentration ([Ca^2+^]_er_) and mitochondrial Ca^2+^ concentration ([Ca^2+^]_m_), the astrocytes were co-transfected with the plasmids pCMV R-CEPIA1er and pCMV CEPIA2mt using Lipofectamine 2000 (Invitrogen) as described (Rao et al., 2015). These plasmids are calcium-measuring organelle-entrapped protein indicators and were a gift from Masamitsu Iino (Addgene plasmids #58216 and #58218, respectively; Suzuki et al., 2014). Imaging was performed in NES solution 24 h after transfection. Fluorescence images were acquired with Zeiss Cell Observer Spinning Disk Confocal microscope, LD LCI Plan-Apochromat 25×/0.8W objective, Zen 2012 Blue software and Hamamatsu ImagEM EM-CCD camera. Images were captured at 1-s intervals for up to 2 min in 37°C and 5% CO_2_. The following excitation/emission wavelengths were used: pCMV CEPIA2mt (excitation 488 nm, emission 525/50 nm) and pCMV R-CEPIA1er (excitation 561 nm, emission 629/62 nm). Ca^2+^-insensitive fluorescence was subtracted from each wavelength before calculations to normalize fluorescence values. The values were then plotted against time and shown as F_1_/F_0_ (F_0_, at rest; F_1_, after administration of ATP, background subtracted). The peak fluorescence and peak time were measured for each plot. The change in intraorganellar free calcium concentration ([Ca^2+^]_er_ and [Ca^2+^]_m_) was assessed for each individual cell as peak fluorescence from the corresponding plot ([1 – F_1_/F_0_, r.u.], and [F_1_/F_0_ – 1, r.u.], respectively).

### HIF1α and HIF2α siRNA transfection

The sequences of siRNA targeting mouse HIF1α and HIF2α were predesigned by Sigma (RNAi ID: SASI_Mm01_00070476 and SASI_Mm01_00070480 for HIF1α, SASI_Mm01_00144144 and SASI_Mm02_00317873 for HIF2α). Cyanine 5 fluorescent group was added to 5′ end of the sense strand. MISSION siRNA Fluorescent Universal Negative Control #1, Cyanine 5 (SIC005, Sigma) was used as a negative control. Astrocytes were transfected with the siRNA using X-tremeGENE siRNA Transfection reagent (Sigma) according to the manufacturer’s instructions. After treatment with siRNA, the cells were incubated at 37°C with 5% CO_2_/95% air for further 24 h. At this time point, the majority of cells were Cyanine 5 positive.

### Total internal reflection fluorescence (TIRF) microscopy

Vesicular exocytosis in primary astrocytes was studied by an optical method (Miesenböck et al., 1998; Sankaranarayanan et al., 2000) by imaging of a superecliptic Syb2-pHluorin. The Syb2-pHluorin plasmid was kindly provided by Prof. Gero Miesenböck. Astrocytes plated on cell culture dishes with a glass bottom (65,000 cells/cm^2^) were transfected with the Syb2-pHluorin plasmid using Lipofectamine 2000 (Invitrogen), and 24 h later, time-lapse TIRF imaging was performed. During imaging cells were incubated in an environmental control system set to 37°C and 5% CO_2_ in the NES-buffer. Zeiss Cell Observer Spinning Disk Confocal microscope equipped with Laser TIRF3 module and alpha Plan-Apochromat 63×/1.46 objective (Carl Zeiss) was used for TIRF imaging in combination with Hamamatsu ORCA-R2 camera (Hamamatsu) controlled by Zen 2012 Blue software. Excitation laser wavelength was 488 nm, and images were acquired through a 525/31-nm bandpass filter at the rate of one image per second. When focusing on the cell, multiple fusion/release events of Syb2-pHluorin-positive vesicles over time were observed as a sudden appearance of spot-like fluorescent signal in evanescent field followed by diffusion of signal in the vicinity. We quantified automatically the number of Syb2-pHluorin fluorescent spots by thresholding the signals that were significantly brighter than the cellular background per time frame in TIRF movies. Analysis of TIRF movies was performed using Zen 2012 Blue software and particle analyzer algorithm implemented as a plugin in Fiji (Schindelin et al., 2012). Data are presented as the number of exocytotic events per μm^2^ of cellular surface over time, typically 2 min. When indicated, data are normalized by resting levels, and are presented as ratio between the number of evoked exocytotic events per μm^2^ and the number of baseline exocytotic events per μm^2^ over time. For statistical analysis of both the raw and normalized data, the area under the curve was counted using the GraphPad Prizm software.

### Quantification of intracellular ATP level

Whole-cell lysates from cultured primary cortical astrocytes were prepared in a 50 mm Tris-HCl, pH 8.0, 50 mm NaCl, 1% Triton X-100, and 1 mm dithiothreitol lysis buffer supplemented with protease inhibitor cocktail (Roche). The ATP amount was quantified in aliquots of 2.5 μg of protein using ATP determination kit (Invitrogen) according to the manufacturer’s instructions. Luminescence was measured using Infinite M1000 Pro multi-mode microplate reader (Tecan) and ATP concentrations were calculated according to the manufacturer’s instructions. ATP standard curves were established in each experiment.

### Determination of ATPase activity

Whole-cell lysates were prepared from the primary cortical astrocytes as described above for the ATP quantification. Contamination with inorganic phosphate (Pi) was removed via incubation of the lysate with Pi Bind resin (Innova Biosciences) for 2 h at +4°C. ATPase activity was quantified in aliquots of 10 μg of protein using ATPase assay kit (Innova Biosciences) according to the manufacturer’s instructions. The amount of Pi released was quantified colorimetrically at 630 nm using Infinite M1000 Pro multi-mode microplate reader (Tecan). Pi standard curve was established in each experiment.

### Monitoring of intracellular oxygen content

Intracellular oxygen was assessed using the oxygen-sensitive probe MitoXpress-Intra (LuxelBiosciences). The measurement is based on the ability of O_2_ to quench the emission of the probe, which is taken by endocytosis. Cultured primary cortical astrocytes at full confluence were loaded with MitoXpress-Intra (10 μg/ml) and incubated for further 20 h either in normoxic or OGD conditions. Intracellular O_2_ was then measured using the time-resolved fluorescence mode of the FLUOstar Omega microplate reader (BMG Labtech) according to manufacturer’s instructions, with excitation performed at 340 nm and emission collected at 655 nm. Phosphorescent intensities were measured at delay times of 30 and 70 ms. The ratio of these intensities was subsequently converted into oxygen content in cellular monolayer using the plate reader software MARS with predefined templates.

### Analysis of mitochondrial membrane potential

Mitochondrial membrane potential was measured based on the accumulation of tetramethylrhodamine methyl ester (TMRM) fluorescence using FLUOstar Omega microplate reader and the Mitochondrial Membrane Potential Assay kit (Cell Signaling) according to the manufacturer’s instructions. TMRM is a cell membrane permeable cationic dye, which accumulates electrophoretically into mitochondria in response to the negative mitochondrial Δψ (Ehrenberg et al., 1988). Primary cortical astrocytes were loaded with 150 nm TMRM (Sigma) for 5 min in an assay buffer containing 80 mm NaCl, 75 mm KCl, 25 mm d-glucose, and 25 mm HEPES, pH 7.4. Fluorescence was measured on the plate reader at excitation 544 nm and emission 590 nm. In order to control for plasma membrane potential variations, each assay was performed in parallel as above with a 15-min preincubation with 10 μm carbonyl cyanide 3-chlorophenylhydrazone (CCCP; Sigma). All data were expressed as the total TMRM fluorescence minus the CCCP treated TMRM fluorescence.

### Blue native (BN) electrophoresis

Mitochondrial protein complex samples for BN-PAGE were prepared from cultured primary cortical astrocytes as previously described (Nijtmans et al., 2002). Digitonin (2 mg/ml)-treated cell pellets were solubilized in 1.5 m aminocaproic acid, 50 mm Bis-Tris-HCl, pH 7.0, and 1% dodecylmaltoside. The samples were incubated on ice for 15 min and centrifuged at 20,000 × *g* for 20 min to remove insolubilized material. Supernatants containing the mitochondrial protein complexes were collected. BN–PAGE electrophoresis and blotting were performed as previously described (Ugalde et al., 2004). Briefly, 20-μg samples were combined with 5% Serva blue G and separated on 5–15% gradient acrylamide gel. The proteins were transferred to a nitrocellulose membrane by semi-dry protein transfer. Western blotting was performed using antibodies against NDUFS3 (Abcam, ab14711), ATP5A (Abcam, ab14748), UQCRC2 (Abcam, ab14745), COX I (Molecular probes, A6403), and SDHA (Abcam, ab14715).

### Transmission electron microscopy (TEM)

TEM was conducted as previously described (Konzack et al., 2015). The primary cortical astrocytes were fixed in 1% glutaraldehyde and 4% formaldehyde mixture in 0.1 m phosphate buffer for 10 min. The cells were detached, and fixation was continued for 1 h. After fixation, the cells were centrifuged, immersed in 2% agarose in distilled water, postfixed in 1% osmium tetroxide, dehydrated in acetone, and embedded in Epon LX 112 (Ladd Research Industries). Thin sections were cut with a Leica Ultracut UCT ultramicrotome, stained in uranyl acetate and lead citrate, and examined in a Tecnai G2 Spirit TEM (FEI Europe). Images were captured by using a Quemesa CCD camera (Olympus Soft Imaging Solutions GmbH) and analyzed with a Tecnai G2 Spirit 120 kV TEM with Veleta and Quemesa CCD cameras and a Philips CM100 equipped with CCD camera 23.

### Mitochondrial morphometry

TEM images were analyzed using iTEM software. Mitochondrial morphologic characteristics including the number of mitochondria per square area of the cell, area of individual mitochondrion, length or aspect ratio (the ratio between the major and minor axes of the ellipse equivalent to the mitochondrion), degree of branching or form factor [defined as (P_m_^2^)/(4πA_m_), where P_m_ is the length of mitochondrial outline and Am is the area of mitochondrion] were quantified as previously described (Mortiboys et al., 2008). In addition, the number of electron-lucent (clear) coated and uncoated vesicular structures per square area of the cell, as well as average area of these vesicles were quantified.

### Immunostaining for Tom20

Primary cortical astrocytes grown on poly-d-lysine-coated coverslips were fixed with 20% methanol for 7 min and permeabilized with 0.1% Triton X‐100/PBS for 15 min. The cells were then incubated in 5% bovine serum albumin (BSA)/PBS blocking solution for 30 min and subsequently incubated with a Tom20 antibody (Cell Signaling, 42406S) diluted in blocking solution overnight at +4°C. After washing, the fluorescent Cy3-conjugated secondary antibody (Jackson ImmunoResearch) were diluted in 1% BSA/PBS and applied for 1 h at room temperature. Immunofluorescence data were obtained using Zeiss Axio Scope.A1 fluorescence microscope with a Zeiss AxioCam MRm Camera (Carl Zeiss) equipped with Zen 2011 Blue software. To estimate mitochondria distribution in the cells the number of cells with clear mitochondrial staining in distal processes (phenotype 1) and the number of cells with distal processes virtually devoid of any staining (phenotype 2) were quantified as percent to the total number of cells.

### Experimental design and statistical analysis

Experimental design and details on the number of animals and samples used in each individual experiment are specified in the figure legends. Data, expressed as mean ± SEM, were analyzed using the GraphPad Prism statistical analysis software. The data were checked for Gaussian distribution using the D'Agostino–Pearson omnibus normality test or Shapiro–Wilk normality test. In case of comparison between two groups, unpaired two-tailed Student’s *t* test was performed. When comparisons were done between three or more groups, the data were analyzed using one-way ANOVA test with subsequent *post hoc* tests. Values of *p* < 0.05 were considered statistically significant.

## Results

### Expression of several genes involved in calcium signaling, in particular certain calcium sequestering ATPases, is altered in P4H-TM KO mice

**Figure 1.**
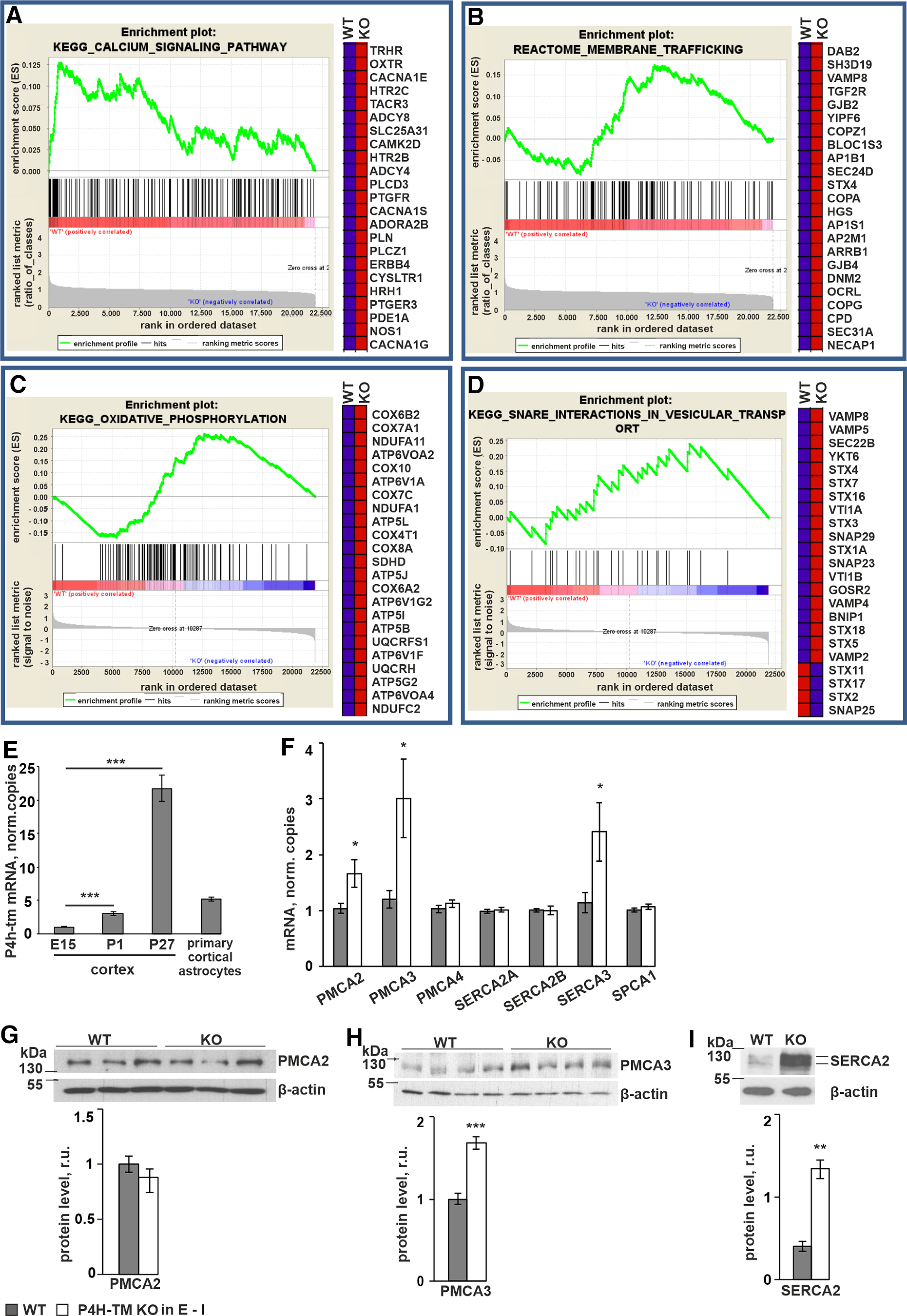
Microarray data suggest that P4H-TM regulates calcium signaling. ***A–D***, Male P4H-TM KO mice and their WT littermates were killed at the age of 2.5 months, and cortexes were collected for microarray analysis. Gene expression was compared between WT and KO and significantly enriched datasets were selected according to GSEA with default settings, i.e., *p* < 0.05. Red color represents upregulated and blue color downregulated genes. Four individual mice were used per genotype in the experiment. Enrichment scores with a ranked list metrics (left) and heat map of 23 leading edge genes (right) are shown for the following biological pathways: (***A***) GSEA for calcium signaling pathway, (***B***) membrane trafficking, (***C***) oxidative phosphorylation, and (***D***) SNARE interactions in vesicular transport. ***E***, WT mice were killed either for dissection of cortexes or for isolation of primary cortical astrocytes. Cortexes were isolated from E15, P1, and P27 mice, while primary astrocytes were isolated from P1 to P2 mice and cultured for 10 d for *P4h-tm* mRNA analysis by qRT-PCR; *n* = 4 mice for tissue dissection per time point and *n* = 3 individual cultures per genotype, 6 mice per genotype. ***F***, qRT-PCR analysis of mRNA expression of different calcium pumps in primary cortical astrocytes. Plasma membrane calcium ATPase 2 (PMCA2) and 3 (PMCA3) and ER calcium ATPase 3 (SERCA3) mRNAs were found to be upregulated in P4H-TM KO versus WT cells; *n* = 9–12, 4 individual cultures per genotype with two to three technical replicates each, 12 mice per genotype. ***G–I***, Western blot analysis of expression of PMCA2 (***G***), PMCA3 (***H***), and SERCA2 (***I***) proteins in P4H-TM KO versus WT primary cortical astrocytes. Representative blots are shown (upper panels) and the intensity of bands is quantitated by densitometry (lower panels), *n* = 3–4 individual cultures per genotype, 6–8 mice per genotype. β-Actin represents a loading control in ***G***, ***I***, while in ***H***, it represents a control of equal protein amount in the samples, because the high amount of protein needed for PMCA3 detection resulted in overloading of β-actin, and it therefore could not be analyzed from the same gel. Data information: data (***E–I***) are presented as mean ± SEM; *p < 0.05, **p < 0.01, ***p < 0.001 (Student’s *t* test). r.u., relative unit.

We have recently shown high abundance of P4H-TM expression in the cortex, amygdala, hippocampus and hypothalamus in adult mice (Leinonen et al., 2016). To study the functional role of P4H-TM in the brain, we first performed microarray experiments of cortical tissue isolated from WT and P4H-TM KO (*P4h-tm*^−/−^) mice. Comparison of the expression data by GSEA software revealed significant changes in calcium signaling, membrane trafficking, oxidative phosphorylation, and SNARE interactions in vesicular transport pathways (Fig. 1*A–D*). Based on the GSEA analysis, we hypothesized that P4H-TM is involved in the regulation of active vesicular transport via calcium signaling in the brain. qRT-PCR analyses showed upregulation of P4H-TM mRNA expression over time in mouse cortical tissue from embryonic day (E)15 to one month of age (Fig. 1*E*). We chose to study the role of P4H-TM further in primary astrocyte cultures established from postnatal day (P)1 to P2 cortexes. Expression of P4H-TM mRNA in these cells was verified by qRT-PCR (Fig. 1*E*), confirming their suitability for functional studies of P4H-TM.

As the microarray data indicated alterations in the expression of genes involved in calcium signaling in the *P4h-tm*^−/−^ mouse cortex relative to WT (Fig. 1*A*), including several Ca^2+^ transporting ATPases, we next analyzed the expression of various Ca^2+^ ATPases in the cultured astrocytes by qRT-PCR. The results showed mRNA upregulation of the plasma membrane Ca^2+^ ATPases PMCA2 and PMCA3 and the sarcoplasmic/ER Ca^2+^ ATPase SERCA3 isoform in *P4h-tm*^−/−^ astrocytes (Fig. 1*F*). No difference in the expression of SPCA1 or PMCA4, SERCA2A or SERCA2B mRNA was observed between the genotypes (Fig. 1*F*). At protein level, upregulation of PMCA3, but not PMCA2, was confirmed by Western blotting in *P4h-tm*^−/−^ cells relative to WT (Fig. 1*G*,*H*). We analyzed expression of SERCA by antibodies against SERCA2 and SERCA3 and detected upregulation of SERCA2 protein in *P4h-tm*^−/−^ cells relative to control despite no changes in the mRNA level between the genotypes (Fig. 1*F*,*I*). Variable and nonreproducible results depending on the antibody source were obtained for SERCA3 expression and thus conclusions of SERCA3 protein expression level could not be made.

### Receptor-mediated and store-operated calcium entry (SOCE) and the ER calcium content is affected in *P4h-tm*^−/−^ astrocytes

**Figure 2.**
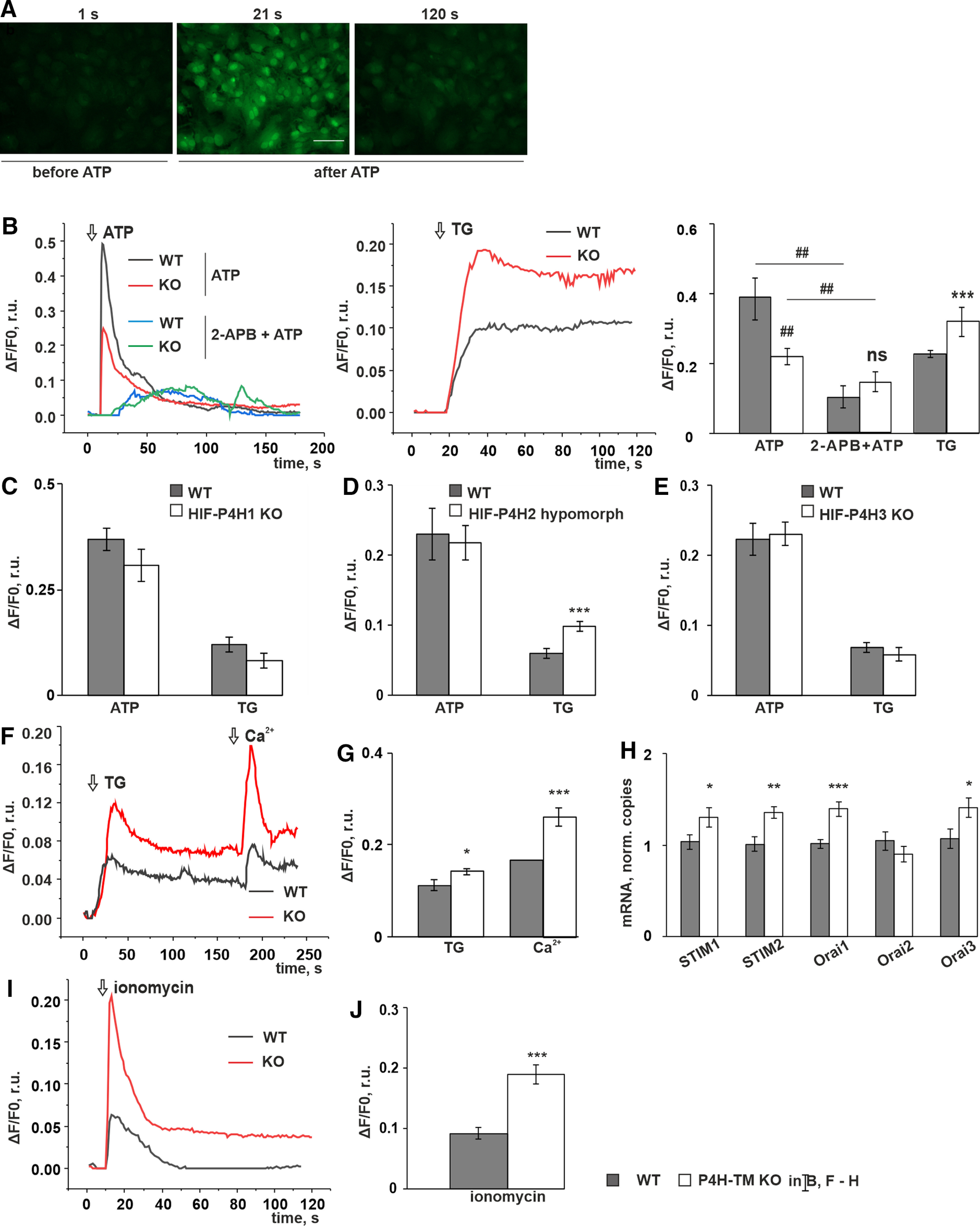
P4H-TM inactivation affects agonist-evoked changes in cytosolic calcium concentrations. ***A–F***, ***H***, Primary cortical astrocytes were isolated from P4H-TM KO and WT mice and loaded with Fluo-4. Representative traces of P4H-TM KO and WT astrocytes are shown on the graphs. Changes in cytosolic calcium [Ca^2+^]i peak or plateau amplitudes are shown in the diagrams. ***A–E***, The increase in [Ca^2+^]i was evoked by either ATP (100 μm) or TG (10 μm) in a calcium-containing buffer. ***A***, Fluorescence of the Fluo-4 calcium indicator captured by a spinning disk confocal microscope. Panels show a time point before the treatment and two time points after stimulation with ATP and illustrate a massive increase in the signal a few seconds after addition of ATP, followed by a subsequent decay. Scale bar: 60 μm. ***B***, Changes in [Ca^2+^]i in P4H-TM KO versus WT astrocytes on treatment with ATP, 2-APB + ATP, and TG. In 2-APB + ATP the cells were preincubated with 2-APB (25 μm; inhibitor of IP3R) 2 min before ATP addition; *n* = 46 cells per genotype in ATP, *n* = 14 cells per genotype in 2-APB-2 + ATP and *n* = 46–61 cells per genotype in TG, 4 mice per genotype. ***C–E***, Changes in [Ca^2+^]i in HIF-P4H1 KO (***C***, *n* = 20 cells in ATP and *n* = 13–18 cells in TG per genotype, 3 mice per genotype), HIF-P4H2 hypomorph (***D***, *n* = 15–19 cells per genotype, 3 mice per genotype), and HIF-P4H3 KO (***E***, *n* = 18–25 cells per genotype, 3 mice per genotype) versus corresponding WT after stimulation with either ATP or TG. ***F,G***, TG-evoked changes in [Ca^2+^]i in P4H-TM KO and WT astrocytes in EGTA (2 mm)-containing buffer and the effect of readdition of 20 mm calcium, *n* = 31–35 cells per genotype, 4 mice per genotype. ***H***, qRT-PCR analysis of *Stim* and *Orai* isoform mRNA levels in P4H-TM KO and WT astrocytes, *n* = 7–11, 3–4 individual cultures per genotype with one to three technical replicates each, 9–12 mice per genotype. ***I,J***, Changes in [Ca^2+^]i in P4H-TM KO and WT astrocytes evoked by ionomycin (1 μm) in the presence of EGTA (2 mm) in the buffer, *n* = 23–31 cells per genotype, 3 mice per genotype. Data information: data are presented as mean ± SEM in ***B–J***; ##*p* < 0.01 by Tukey’s HSD test after one-way ANOVA method for multiple comparisons; *p < 0.05, **p < 0.01, ***p < 0.001 by Student’s *t* test. r.u., relative unit. ns, not significant.

We next studied the effect of P4H-TM on calcium signaling by monitoring the changes in intracellular free calcium concentration ([Ca^2+^]_i_) in WT and *P4h-tm*^−/−^ astrocytes loaded with the calcium indicator Fluo-4. The increase in [Ca^2+^]_i_ can be evoked in astrocytes via two different mechanisms: receptor-operated calcium entry (ROCE) and SOCE (Berridge et al., 2000; Clapham, 2007; Rivera et al., 2016; Papanikolaou et al., 2017). To investigate the possibility that P4H-TM affects ROCE, we stimulated the cells with ATP (King et al., 1996; Fischer et al., 2009; Fig. 2*A*). The ATP-evoked calcium response was substantially attenuated in the *P4h-tm*^−/−^ cells relative to WT (Fig. 2*B*). The difference in the response was abolished by addition of 2-APB, an inhibitor of ROCE (Bootman et al., 2002; Fig. 2*B*). Treatment of the cells with TG, a potent SERCA inhibitor that depletes intracellular calcium stores and evokes substantial SOCE in astrocytes (Calloway et al., 2010), showed a significantly higher response in the *P4h-tm*^−/−^ astrocytes (Fig. 2*B*), suggesting increased SOCE in *P4h-tm*^−/−^ astrocytes relative to WT.

To determine whether the observed effects were a specific outcome of the KO of P4H-TM or whether the other HIF regulating P4Hs contribute to it, we next studied calcium entry in astrocytes isolated from *Hif-p4h-1*^−/−^, *Hif-p4h-3*^−/−^, and *Hif-p4h-2* hypomorph mice. No differences were detected in the ATP-induced calcium response in these mutant astrocytes in comparison to WT (Fig. 2*C–E*), suggesting that the effect of P4H-TM on ROCE is unique among the HIF regulating P4Hs. However, SOCE was affected in *Hif-p4h-2* hypomorph astrocytes, but not in *Hif-p4h-1*^−/−^ or *Hif-p4h-3*^−/−^ astrocytes (Fig. 2*C–E*), HIF-P4H2 hypomorph cells having a significantly higher response to TG (Fig. 2*D*). The HIF-P4H-2 mRNA expression level in the primary *Hif-p4h-2* hypomorph astrocytes was ∼20% of that in WT astrocytes (0.21 ± 0.02 r.u. in HIF-P4H-2 hypomorph cells vs 1.00 ± 0.08 r.u. in WT cells, *n* = 3 individual cultures isolated from six mice per genotype, cells from two mice pooled per culture, *p* < 0.001 by Student’s *t* test, qRT-PCR data). P4H-TM and HIF-P4H2 thus apparently share some overlapping molecular mechanisms to regulate SOCE and the role of HIF-P4H-2 in the regulation of calcium signaling should be a topic for further investigation.

To dissect further the effect of P4H-TM KO on SOCE, we monitored calcium influx in Fluo-4-loaded cells after depleting ER calcium stores with TG in EGTA-containing buffer followed by superfusion with calcium-containing buffer (Berridge et al., 2000; Papanikolaou et al., 2017). Under these experimental conditions, virtually all extracellular calcium is chelated, and the TG-evoked increase in [Ca^2+^]_i_ is generated by depletion of intracellular stores only and is thus proportional to the ER calcium content. A subsequent readdition of calcium induces an increase in [Ca^2+^]_i_ exclusively by massive influx through the SOCE channels. The *P4h-tm*^−/−^ astrocytes had a significantly higher TG-evoked rise in [Ca^2+^]_i_ relative to WT (Fig. 2*F, G*). This suggested that the calcium content within the ER is higher in *P4h-tm*^−/−^ cells. The signal from readdition of calcium was higher in the *P4h-tm*^−/−^ astrocytes (Fig. 2*F, G*), confirming enhanced SOCE in P4H-TM KO relative to WT cells. qRT-PCR analysis of the mRNA expression of the main proteins involved in SOCE (Gao et al., 2016; Kwon et al., 2017) showed that mRNA levels for STIM1, STIM2, ORAI1 and ORAI3 were upregulated in the *P4h-tm*^−/−^ astrocytes relative to WT (Fig. 2*H*), being in line with the live-cell calcium imaging data.

To further investigate the possibility that P4H-TM deficiency affects the calcium content of the ER, we stimulated the cells in the presence of EGTA with ionomycin, a potent, highly selective calcium ionophore, that induces an increase in [Ca^2+^]_i_ primarily by physicochemical translocation of calcium through the lipid bilayer of intracellular stores with only minor contribution of the ROCE pathway (McCollum et al., 2004; Müller et al., 2013). Since the translocation occurs according to the calcium gradient, the peak of the response to ionomycin is proportional to the ER calcium content. The response to ionomycin was significantly higher in the *P4h-tm*^−/−^ astrocytes relative to WT (Fig. 2*I, J*). Taken together, our results show that the ER calcium content is higher in *P4h-tm*^−/−^ astrocytes, and thus cannot explain the observed lower ROCE (Fig. 2*B*) in the *P4h-tm*^−/−^ astrocytes relative to WT, which therefore must result from some other mechanism.

### Re-uptake of calcium by mitochondria on ATP treatment is significantly higher in P4H-TM KO astrocytes

**Figure 3.**
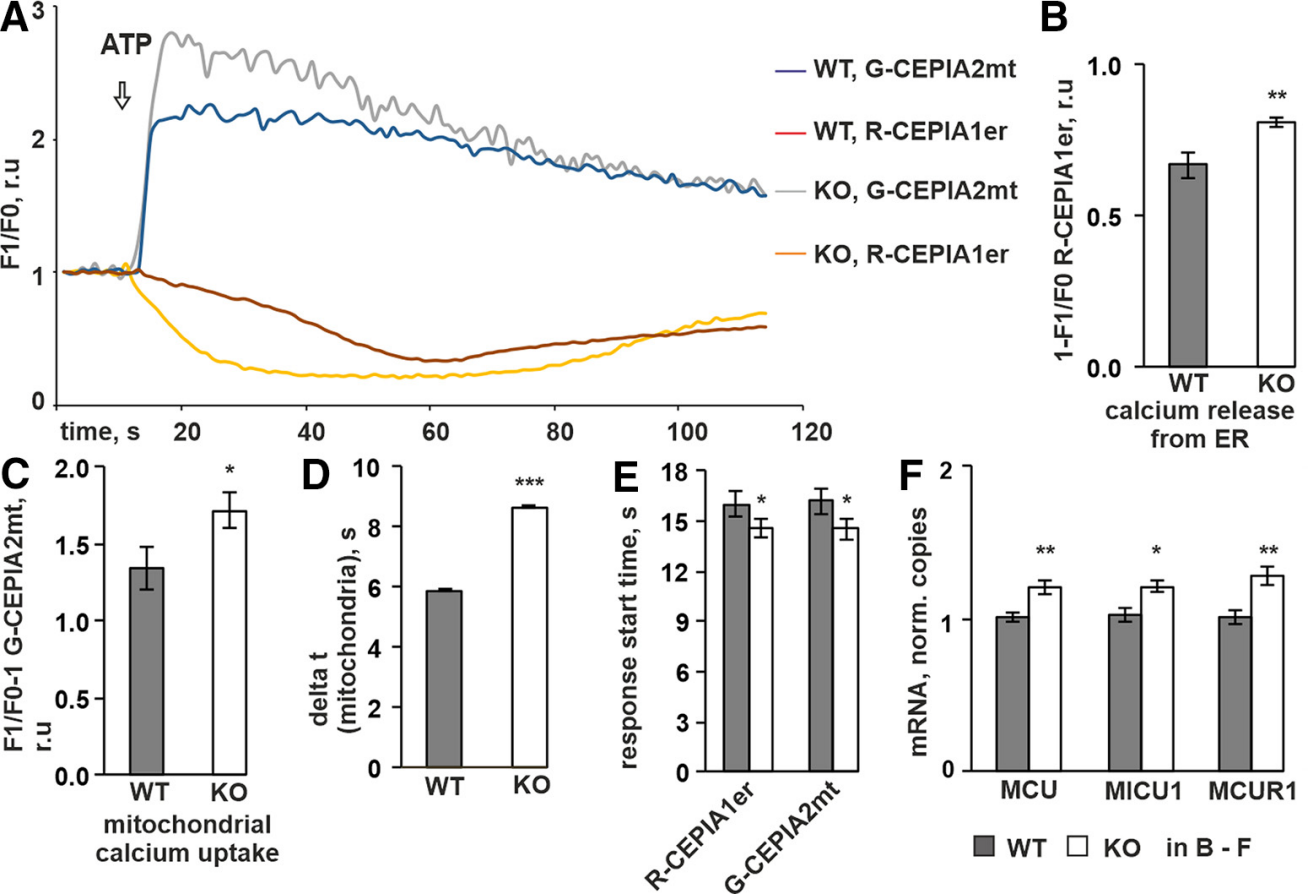
P4H-TM KO affects mitochondrial and ER calcium dynamics in response to ATP. ***A–E***, P4H-TM KO and WT primary cortical astrocytes were co-transfected with G-CEPIA2mt (to measure mitochondrial calcium concentration) and R-CEPIA1er (to measure changes in ER calcium concentration), and fluorescence intensity was recorded during time-lapse imaging in a calcium-containing buffer. The astrocytes were stimulated with 100 μm ATP. Analysis of time-lapse images was done on *n* = 26 cells per genotype, 6 mice per genotype. ***A***, Representative traces of the ATP-induced changes in ER and mitochondrial calcium concentrations. Time course of F1/F0 ER calcium signal indicates red fluorescence decrease on ATP treatment proportional to decrease of free calcium amount inside the ER. Time course of F1/F0 mitochondrial calcium signal indicates sudden increase in green fluorescence on ATP treatment proportional to rapid accumulation of calcium inside mitochondria. In the majority of cells, decrease in ER calcium signal preceded increase in mitochondria calcium signal. ***B–D***, Amplitudes of ER and mitochondria calcium response to ATP were defined as maximum change in F1/F0 (fluorescence intensity after ATP application normalized by the resting value) within a 2-min time window after ATP application. ***B***, The ATP-evoked release of ER calcium was higher in P4H-TM KO astrocytes. ***C***, The ATP-evoked increase in mitochondrial calcium was higher in P4H-TM KO astrocytes. ***D***, Time necessary for mitochondria to reach the maximum amplitude of calcium response starting from the resting level. ***E***, Time point when the red fluorescence (ER signal) starts to decrease and the green fluorescence (mitochondrial signal) starts to increase on ATP treatment. The data indicate that P4H-TM KO astrocytes overall react faster to ATP stimulation than WT astrocytes. ***F***, qRT-PCR analysis of mRNA levels for the mitochondrial uniporter channel complex components MCU, MICU1, and MCUR1 in P4H-TM KO and WT astrocytes, *n* = 8–12, 3–4 individual cultures per genotype with two to three technical replicates each, 9–12 mice per genotype. Data information: data are presented as mean ± SEM in ***B–F***; *p < 0.05, **p < 0.01, ***p < 0.001 by Student’s *t* test. r.u., relative unit.

The reduced ATP-evoked ROCE in *P4h-tm*^−/−^ astrocytes relative to WT is intriguing, as based on the higher ER calcium content in the *P4h-tm*^−/−^ astrocytes when compared with WT, an opposite effect could be expected. Nevertheless, the [Ca^2+^]_i_ response on ROCE is a net result of calcium entry from the extracellular milieu, and both the release of calcium from intracellular stores and uptake of calcium by other organelles, such as mitochondria, acting as calcium sinks (Filadi et al., 2017). Therefore, we next studied mitochondrial calcium uptake in *P4h-tm*^−/−^ astrocytes by analyzing simultaneously calcium release from the ER and calcium accumulation within mitochondria using genetically-encoded calcium indicators (GECIs) entrapped in ER and mitochondria, respectively (Suzuki et al., 2014, 2016). Astrocytes were co-transfected with ER-targeted red fluorescent R-CEPIA1er and mitochondria-targeted green-fluorescent G-CEPIA2mt, followed by live-cell imaging on ATP stimulation at 24 h posttransfection. Changes in red and green fluorescent signal proportional to changes in free calcium concentration inside ER and mitochondria, [Ca^2+^]_er_ and [Ca^2+^]_m_, respectively, were calculated, and an ATP-induced decrease in [Ca^2+^]_er_ accompanied with an increase in [Ca^2+^]_m_ was observed (Fig. 3*A*). The maximum amplitude of the ER response was significantly higher in *P4h-tm*^−/−^ astrocytes than in WT (Fig. 3*A*,*B*). Since [Ca^2 +^]_er_ at the resting state was used as a normalization value, this result is in accordance with the higher calcium content inside the ER in *P4h-tm*^−/−^ cells. As is evident from the fluorescent plot, the kinetics of calcium release from the ER was faster in P4H-TM KO cells (Fig. 3*A*). The maximum amplitude of the mitochondria response in *P4h-tm*^−/−^ astrocytes was likewise more profound than in the WT cells (Fig. 3*A*,*C*), indicating higher mitochondrial uptake of calcium in the mutant cells. Furthermore, the time to reach a maximum response was significantly longer in the mutant cells in comparison to WT cells (Fig. 3*D*). Interestingly, both the ER and mitochondria responses to addition of ATP started significantly faster in *P4h-tm*^−/−^ astrocytes in comparison to WT cells (Fig. 3*E*, 1.43 and 1.61 s faster, respectively). The higher mRNA levels for mitochondrial calcium uniporter (MCU), mitochondrial calcium uptake 1 (MICU1), and MCU regulator 1 (MCUR1; Fig. 3*F*), i.e., the proteins regulating mitochondrial calcium uptake (Perocchi et al., 2010; Filadi et al., 2017), could suggest higher protein expression, and in part explain the higher uptake of calcium in *P4h-tm*^−/−^ astrocytes.

Taken together, P4H-TM KO mitochondria have a higher ATP-induced calcium uptake capacity than the WT cells. Therefore, it is likely that the higher mitochondrial uptake overrides the higher release of calcium from the ER, the net effect being a decrease in the ATP-evoked changes in [Ca^2+^]_i_ in the mutant cells relative to WT cells.

### Expression of calcium leak channels is increased in P4H-TM KO astrocytes

**Figure 4.**
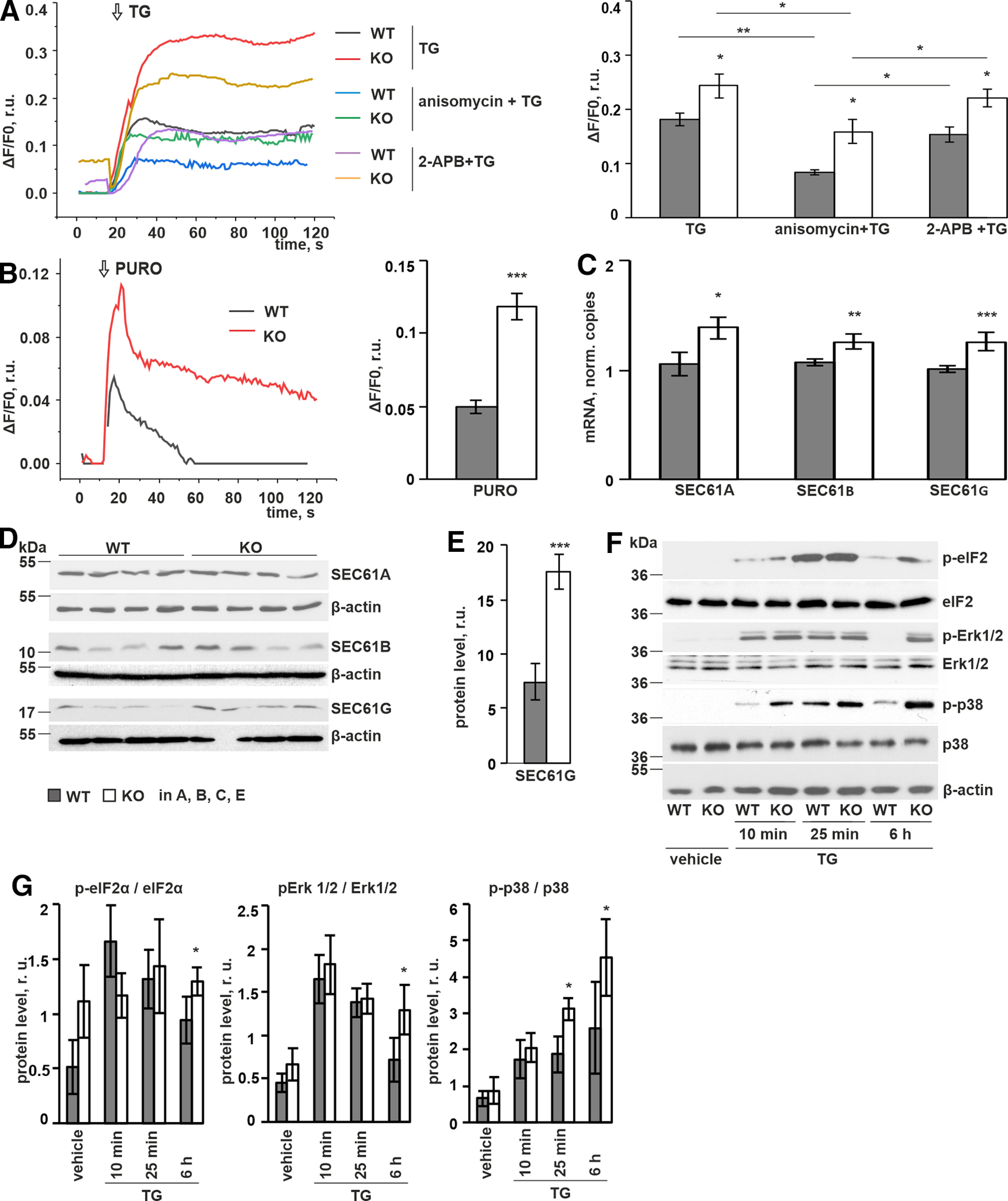
P4H-TM KO affects passive calcium leak through translocon complex and IP3R. ***A***, ***B***, P4H-TM KO and WT primary cortical astrocytes were loaded with Fluo-4, and live cell calcium imaging was performed in the presence of the indicated pharmacological inhibitors. Representative traces of P4H-TM KO and WT astrocytes are shown on the graphs. Changes in cytosolic calcium [Ca^2+^]i peak or plateau amplitudes are shown in the diagrams. ***A***, The increase in [Ca^2+^]i evoked by stimulating the cells with TG (10 μm) was attenuated by the translocon inhibitor anisomycin (200 μm, 10 min before TG), but not by the IP3R inhibitor 2-APB (25 μm, 2 min before TG), *n* = 22–38 cells per genotype, 5 mice per genotype per condition. Imaging was performed in a calcium-containing buffer. ***B***, Application of an inhibitor of translation (puromycin, PURO, 200 μm) induced a higher increase in [Ca^2+^]i in P4H-TM KO astrocytes versus WT in EGTA-containing buffer, *n* = 16 cells per genotype, 3 mice per genotype. ***C***, qRT–PCR analysis of mRNA expression levels for the translocon complex subunits SEC61 A, B, and G in P4H-TM KO and WT astrocytes, *n* = 7–12, 3–4 individual cultures per genotype with one to three technical replicates each, 9–12 mice per genotype. ***D***, ***E***, Western blot analysis of expression of translocon subunits SEC61 A, B, and G, in P4H-TM KO and WT astrocytes. Representative Western blots (***D***) and their quantification, *n* = 4 individual cultures per genotype, 4 mice per genotype (***E***) are shown. β-Actin represents a loading control. ***F***, ***G***, Western blot analysis of phosphorylation of elF2a and Erk1/2 and p38 kinases in P4H-TM KO and WT astrocytes after treatment with 10 μm TG for the indicated time points. Representative Western blots (***F***) of the phospho and total forms are shown. β-Actin is shown to confirm equal protein amount in the samples. Western blot quantification, *n* = 3–4 individual cultures per genotype, 3–4 mice per genotype (***E***) are shown. Data information: data are presented as mean ± SEM in ***A–C***, ***E***. In ***A***: *p < 0.05, **p < 0.01 by Holm test after one-way ANOVA for multiple comparisons. In ***B***, ***C***, ***E***, ***G***: *p < 0.05, **p < 0.01, ***p < 0.001 by Student’s *t* test. r.u., relative unit.

In the resting state, the calcium content of the ER reflects a balance between active uptake by SERCA and passive efflux through leak channels such as translocon during protein translation and inositol trisphosphate receptor (IP3R) in its unstimulated stage in astrocytes (Szlufcik et al., 2006; Lang et al., 2017). We next analyzed calcium leakage by inhibiting SERCA pumps with TG simultaneously with pharmacological inhibition of the leak channels. Our data show that pretreatment with anisomycin, an inhibitor of translation that makes the translocon calcium-impermeable, significantly reduced the TG-induced calcium signal in both P4H-TM KO and WT astrocytes, but the difference between the genotypes remained (Fig. 4*A*). Since anisomycin was added 10 min before TG, the pretreatment time is too short for marked changes in protein levels because of inhibition of translation (Aakalu et al., 2001; Claydon and Beynon, 2012), thus the effect can be attributed at least mostly to the inhibition of calcium permeability of the translocon. On the other hand, treatment with 2-APB in a concentration which was efficient to block the response to ATP (Fig. 2*B*) and did not induce a calcium response by itself, was insufficient to block the TG-induced calcium signal (Fig. 4*A*). This suggests that the translocon complex acts as a main leak channel in cortical astrocytes. We next analyzed the effect of puromycin, an inhibitor of translation that blocks the translocon in a calcium permeable way and evokes luminal calcium leakage exclusively through translocon (Van Coppenolle et al., 2004). A higher puromycin-induced calcium leakage was observed in *P4h-tm*^−/−^ cells relative to WT in EGTA-containing buffer (Fig. 4*B*), indicating higher leakage through translocon. This finding was supported by increased mRNA expression levels for the translocon components SEC61A, SEC61B, and SEC61G (Fig. 4*C*), which was also manifested as a higher protein expression level in the case of SEC61G (Fig. 4*D*,*E*) in *P4h-tm*^−/−^ cells relative to WT.

TG-induced translocon-associated calcium loss is also known to contribute to ER stress response modulation and the resulting Erk-elf2α overactivation has been shown to be associated in astrocytes with a distinct pathogenic reactivity state and decreased secretome (Kamiya et al., 2011; Johnson et al., 2014; Martin-Jiménez et al., 2017; Smith et al., 2020). Therefore, we next analyzed the effect of P4H-TM loss on the induction and persistence of Erk and elF2α phosphorylation under TG treatment. The TG-induced phosphorylation of Erk and elF2α has a temporal pattern, the initial increase in phosphorylation plateauing typically at around 2 h followed by a progressive decrease (Smith et al., 2020). No significant difference between the genotypes was observed in the initial phase of the phosphorylation response in the presence of TG, but the phosphorylation of elF2 and Erk1/2 remained at a higher level in the *P4h-tm*^−/−^ cells at a later time point (6 h) when compared with WT (Fig. 4*F*,*G*). Furthermore, the p38 MAPK pathway is known to respond to various cellular and extracellular stress signals and is activated by TG treatment (Kim et al., 2010; Darling and Cook, 2014; Huang et al., 2014). Therefore, we also studied p38 kinase activation on TG treatment. Higher activation of p38 was evident in the *P4h-tm*^−/−^ when compared with WT cells especially at later time points (Fig. 4*F*,*G*). In conclusion, the data show upregulated leak channel expression and higher passive calcium leakage from the ER to the cytosol as well as changes in temporal kinase phosphorylation patterns, which could indicate enhanced susceptibility to ER stress in *P4h-tm*^−/−^ astrocytes. Detailed analyses of the effects of P4H-TM inactivation on the induction of ER stress with various stress inducers, markers and outcomes should be addressed in future studies.

### Calcium-dependent vesicular exocytosis is decreased in P4H-TM KO astrocytes

**Figure 5.**
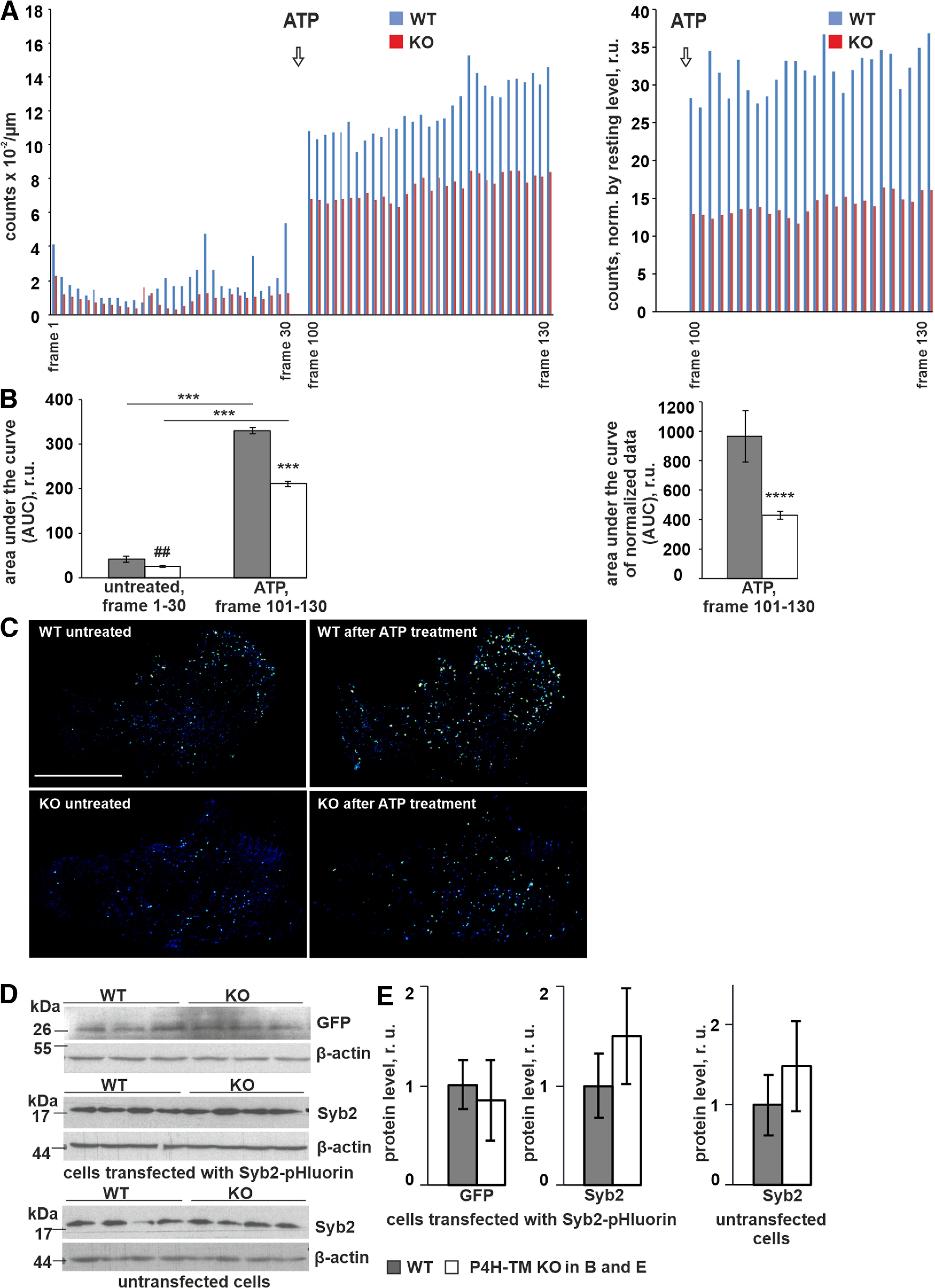
P4H-TM KO affects vesicular exocytosis induced by ATP. Primary cortical astrocytes were transiently transfected with Syb2-pHluorin to label small vesicles. To characterize the time course of vesicle release on the basal plasma membrane, time-lapse image series were generated by taking TIRF images every 1 s over ∼2 min (130 frames). Cells were treated with 100 μm ATP to stimulate calcium-dependent exocytosis, and bright punctate that appeared and then disappeared in the evanescent field was classified as a fusion/release event. ***A***, Analysis of TIRF images was done on 22–25 cells per genotype, 3 individual cultures per genotype, 6 mice per genotype. Number of fusion/release events, which is proportional to the exocytosis rate was quantified using ZEN and then ImageJ software. Each column indicates the average number of fusion/release events in each imaging frame. The number of both spontaneous and ATP-induced fusion/release events (left panel) as well as the number of ATP-induced events normalized by spontaneous signal (right panel) was lower in P4H-TM KO astrocytes when compared with WT. Data are presented as mean. ***B***, Quantification of TIRF data displayed in panel ***A***. The area under the curve (AUC) was calculated using GraphPad Prism software from raw data (left panel) and from normalized data (right panel) from time 0 to 30 s (before ATP treatment, left panel) and from time 100 to 130 s (after ATP treatment, left and right panels) in P4H-TM KO and WT astrocytes, *n* = 22–25 cells per genotype, 3 individual cultures per genotype, 6 mice per genotype. ***C***, Representative background subtracted ratio images of TIRF microscopy showing secretion of Syb2-pHluorin-positive vesicles on the basal plasma membrane. Panels show a time point before and after the ATP treatment and illustrate an overall lower exocytosis rate in P4H-TM KO astrocytes when compared with WT. Scale bar: 20 μm. ***D***, ***E***, Western blot analysis of GFP and Syb2 protein expression in untransfected cells and cells transfected with Syb2-pHluorin as indicated. Representative blots are shown (***D***), and the intensity of bands is quantitated by densitometry (***E***), *n* = 4 individual cultures per genotype, 4 mice per genotype. β-Actin represents a loading control. Data information: data (***B***) are presented as mean ± SEM; ##*p* < 0.01 and ****p < 0.0001 by Student’s *t* test; ***p < 0.001 by Tukey’s HSD test after one-way ANOVA method for multiple comparisons. Data (***E***) are presented as mean ± SEM, not significant by Student’s *t* test, r.u., relative unit.

In response to stimulation with ATP, astrocytes release several chemical substances, termed gliotransmitters, which affect neuronal communication pathways (Parpura and Zorec, 2010; Zorec et al., 2012, 2016). Regulated, calcium-dependent release of gliotransmitters from astrocytes occurs via vesicular exocytosis and one of the integral membrane proteins of the astrocytic secretory vesicles is Syb2 (Zorec et al., 2016). We next studied whether calcium-dependent vesicular exocytosis is affected in *P4h-tm*^−/−^ astrocytes by transfecting the cells with Syb2-pHluorin, a fusion protein consisting of a pH-sensitive GFP mutant fused to the luminal C-terminal end of Syb2 (Miesenböck et al., 1998). Because the lumen of the vesicles is acidic, the fluorescence of Syb2-pHluorin increases on exocytosis because of pH neutralization (Miesenböck et al., 1998). We used TIRF imaging to monitor and quantify the membrane-proximal appearance and disappearance of pHluorin-labeled fluorescent puncta as the indicator of vesicular exocytosis evoked by ATP stimulation on vesicle-cytoplasmic membrane fusion. We also detected some TIRF signal already at baseline before ATP stimulation, but the interpretation of disappearance of fluorescent puncta at baseline is more difficult since clearance of the reporter from the cell surface can occur both via exocytosis and endocytosis. Although treatment of the astrocytes with ATP to stimulate calcium-dependent exocytosis resulted in an overall increase in the pHluorin fluorescence signal, the number of vesicular fusion events was lower in *P4h-tm*^−/−^ cells both at baseline and after ATP stimulation (Fig. 5*A–C*). To make sure that the decreased TIRF signal is not because of differences in Syb2 expression levels we confirmed by Western blotting using anti-GFP and anti-Syb2 antibodies that protein levels of Syb2-pHluorin as well as endogenous Syb2 are equal between genotypes (Fig. 5*D*,*E*). Taking into account that a transient increase in cytosolic calcium levels is sufficient and necessary for the engagement of calcium-sensitive effector proteins of the secretory machinery (Kreft et al., 2004; Zorec et al., 2012), our observation of decreased ATP-evoked vesicular exocytosis in the *P4h-tm*^−/−^ cells relative to WT is well in line with the attenuated ATP-evoked ROCE in these cells.

### Intracellular ATP content is decreased in P4H-TM KO astrocytes

**Figure 6.**
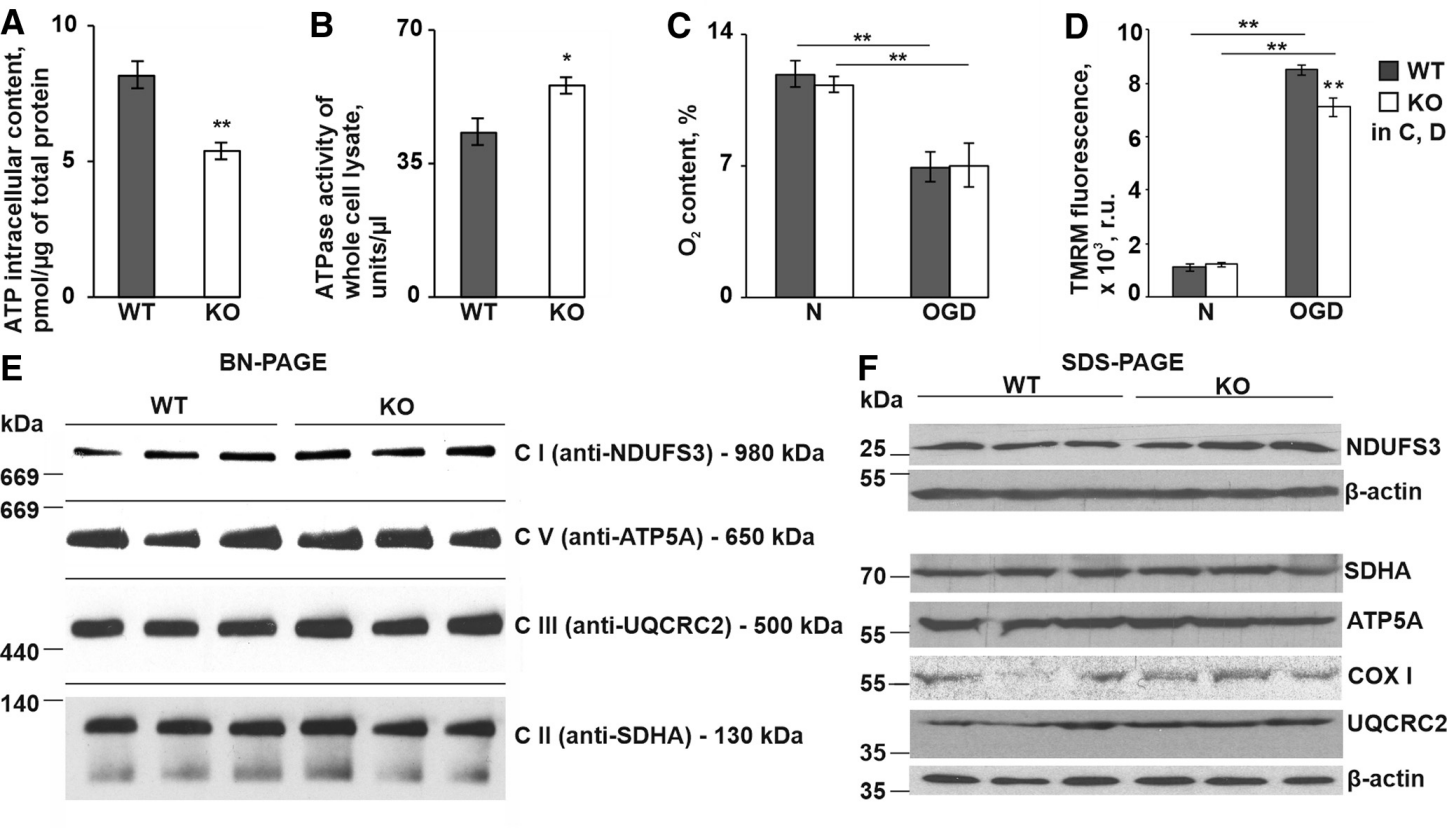
P4H-TM KO affects intracellular ATP content, cellular ATPase activity and mitochondrial membrane potential, without an influence on the amount of mitochondrial respiratory complexes and O_2_ consumption. ***A–F***, Experiments were carried on P4H-TM KO and WT primary cortical astrocytes. ***A***, ATP content in whole-cell lysate, *n* = 5 freshly prepared individual protein lysates per genotype, 5 mice per genotype. ***B***, Comparison of ATPase activity in whole-cell lysate, *n* = 9–10 freshly prepared individual protein lysates, 4–5 mice per genotype. ***C***, ***D***, Astrocytes were incubated in growth medium containing 1 g/l glucose under normoxic conditions (N) or in a medium containing no glucose under hypoxic (1% O_2_) conditions (OGD) for 20 h. ***C***, Intracellular oxygen content within the astrocyte monolayer. Normoxic or hypoxic culture was conducted in the presence of the oxygen-sensitive probe MitoXpress-Intra (10 μg/ml). After 20 h, time resolved fluorescence was measured on FLUOstar Omega microplate reader. Phosphorescent intensity of the probe was converted to O_2_ content using the plate reader software Mars; *n* = 4 individual cultures per genotype, 4 mice per genotype per condition. ***D***, Analysis of mitochondrial membrane potential in living astrocytes. After 20 h of normoxia or OGD astrocytes were loaded with TMRM fluorescent dye (150 nm) for 5 min. Fluorescence was measured on FLUOstar Omega microplate reader. The signal reflects accumulation of TMRM in the mitochondrial membrane and is proportional to membrane potential; *n* = 4 individual cultures per genotype, 4 mice per genotype per condition. ***E***, BN-PAGE analysis of mitochondrial RC complexes in astrocytes. Mitochondrial protein complexes were separated on a 5% – 15% BN-PAGE. Fully assembled Complexes I–V (CI–CV) were assessed using antibodies against the Complex I 39-kDa subunit (NDUFS3), Complex II succinate dehydrogenase complex flavoprotein subunit A (SDHA), Complex III core protein 2 (UQCRC2), Complex IV cytochrome *c* oxidase subunit I (COX I), and Complex V ATP synthase subunit α (ATP5A). The fully assembled Complex IV was under the detection limit. Representative blots of three individual cultures per genotype are shown. ***F***, SDS-PAGE and Western blot analysis of individual RC subunits in whole-cell protein lysates using the antibodies indicated in ***E***. β-Actin represents a loading control. Representative blots of three individual cultures per genotype are shown. Data information: data are presented as mean ± SEM in ***A–D***, *p < 0.05, **p < 0.01 by Student’s *t* test in ***A***, ***B*** and by Tukey’s HSD test after one-way ANOVA method for multiple comparisons in ***C***, ***D***. r.u., relative unit.

As reported above, expression of calcium sequestering ATPases (Fig. 1*F–I*) and mitochondrial uptake of calcium (Fig. 3*A*,*C*) were increased in *P4h-tm*^−/−^ astrocytes. Taking into account the high relative contribution of cellular ATPases to total ATP consumption (Smith et al., 2013) and the importance of ER–mitochondria calcium shuttling for mitochondrial ATP synthesis (Mallilankaraman et al., 2012a,b), we next analyzed intracellular ATP levels and ATPase activity in the *P4h-tm*^−/−^ astrocytes. The ATP content was significantly decreased, while ATPase activity was increased in whole-cell lysates of *P4h-tm*^−/−^ astrocytes relative to WT (Fig. 6*A*,*B*). The data on increased ATPase activity are consistent with the upregulated expression of calcium sequestering ATPases in *P4h-tm*^−/−^ astrocytes.

Previously, it was reported that the ATP synthesis rate in mitochondria correlates with the oxygen consumption rate (Salin et al., 2015), as well as with the mitochondrial membrane potential (Kadenbach et al., 2011). Nevertheless, despite the effect on ATP content, our data revealed no effect of P4H-TM KO on the respiratory capacity of astrocytes, either under normoxic conditions or under OGD (1% O_2_ and no glucose for 20 h; Fig. 6*C*). Interestingly, mitochondrial membrane hyperpolarization, which is known to be induced by OGD as an adaptive attempt of astrocytes to increase ATP production to overcome ischemic stress (Iijima, 2006; Korenić et al., 2014), was impaired in *P4h-tm*^−/−^ astrocytes when compared with WT (Fig. 6*D*). These data indicate that ATP production is likely to be impaired in mitochondria when *P4h-tm*^−/−^ cells are challenged with OGD.

A significant depression of ATP synthesis is seen in isolated brain mitochondria after inhibition of the respiratory chain (RC) Complex I, III, or IV (Davey and Clark, 1996). We therefore next investigated the amount of RC complexes required for oxidative phosphorylation in *P4h-tm*^−/−^ cells. Fresh mitochondria were isolated from digitonin-treated astrocytes and the assembly of OXPHOS complexes was analyzed by BN-PAGE followed by Western blotting. No apparent differences in the amount of assembled Complexes I, II, III, and V were observed between the genotypes (Fig. 6*E*). Unfortunately, we were not able to detect the fully assembled Complex IV by BN-PAGE. However, immunoblotting of individual subunits from all the OXPHOS Complexes I–V, including COXI of Complex IV, indicated no difference between the amounts of protein in whole-cell lysates between the genotypes (Fig. 6*F*). Based on these data, it is unlikely that oxidative phosphorylation is affected in *P4h-tm*^−/−^ cells at least under normoxic conditions and the observed increase in ATPase activity is a likely explanation for the decreased intracellular ATP level in *P4h-tm*^−/−^ astrocytes.

### Ultrastructural analysis of P4H-TM KO astrocytes shows alterations in mitochondria and electron-lucent small vesicles

**Figure 7.**
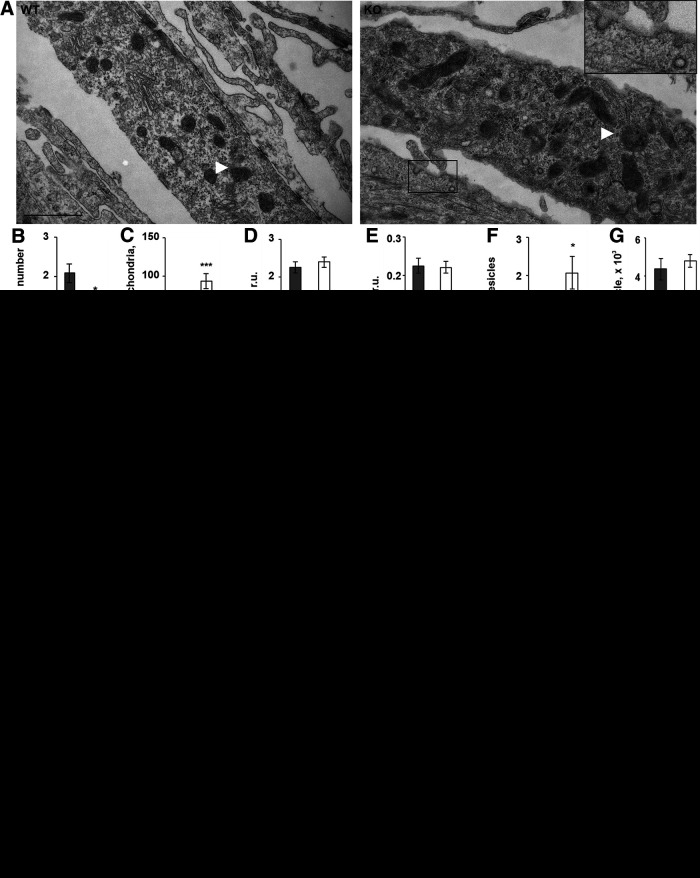
P4H-TM KO affects mitochondrial morphology and leads to accumulation of electron-lucent small vesicles (SLMVs) in the cytosol. ***A–G***, P4H-TM KO and WT astrocytes were analyzed by TEM. Morphometric analysis was performed on randomly selected fields. ***A***, Representative electron micrographs. Multiple mitochondria are visible (white arrows). The boxed area represents a 2.3× magnification: two SLMVs are apparent in the field of view, one of which is releasing its content to the extracellular space. Scale bar: 1 μm. ***B–E***, Morphometric analysis of mitochondrial number per cell area (***B***) and shape (***C–E***), *n* = 7 astrocytes per genotype in ***B***, *n* = 84–103 mitochondria per genotype in ***C–E***, 3 mice per genotype. ***F***, ***G***, Analysis of the number (***F***) and size (***G***) of SLMVs; *n* = 7 astrocytes per genotype in ***F***, *n* = 46 and *n* = 160 SLMVs in WT and KO cells, respectively, in ***G***, 3 mice per genotype. ***H***, ***I***, Primary cortical astrocytes were immunostained with anti-Tom20 antibody for analysis of mitochondrial morphology. Representative images (***H***) demonstrate the observed reduced mitochondrial density within the distal part of the cell processes (white arrows) in the P4H-TM KO astrocytes when compared with WT. Scale bar: 20 μm. The number of cells (***I***) with clear punctate mitochondrial staining in distal processes (phenotype 1) and number of cells with distal processes virtually devoid of any staining (phenotype 2) was quantified as percent to total number of cells, *n* = 6–7 cultures per genotype (corresponds to ∼150 cells analyzed per genotype). Data information: data are presented as mean ± SEM in ***B–G***, ***I***, *p < 0.05, ***p < 0.001 by Student’s *t* test in ***B–G***; *p < 0.05, **p < 0.01 by Tukey’s HSD test after one-way ANOVA method for multiple comparisons in ***I***. r.u., relative unit.

As we observed differences in the vesicular exocytosis and ATP content in *P4h-tm*^−/−^ astrocytes, we next analyzed the number and morphology of mitochondria and vesicles by TEM (Fig. 7*A*). Our data revealed a decrease in the number of mitochondria per area (Fig. 7*B*) accompanied with enlargement of individual mitochondria (Fig. 7*C*) in *P4h-tm*^−/−^ cells, with no effect on mitochondria length or degree of branching (Fig. 7*D*,*E*). The reduced number of mitochondria, but with increased size, is in accordance with the observed equal total OXPHOS subunit protein amount in both genotypes (Fig. 6*F*). In addition, immunostaining for Tom20 in the mitochondrial outer membrane showed that *P4h-tm*^−/−^ astrocytes were frequently essentially devoid of mitochondria in the distal cellular parts/processes (Fig. 7*H*,*I*), indicating changes in the distribution of mitochondria between the genotypes.

Analysis of electron-lucent small vesicular structures (SLMVs; Fig. 7*A*) showed that their number was significantly higher in *P4h-tm*^−/−^ astrocytes than in the WT (Fig. 7*F*), while no difference existed in the vesicle size between the genotypes (Fig. 7*G*). The majority of the vesicles was localized proximal to the plasma membrane and the average diameter was ∼74 nm^2^, which is in the size range (30–100 nm) reported for SLMVs (Vardjan and Zorec, 2015) that are known to contribute to the secretory vesicle population in astrocytes (Montana et al., 2006; Parpura and Zorec, 2010). The observed increased accumulation of SLMVs in the *P4h-tm*^−/−^ cells is likely to be a consequence of the less frequent exocytotic events (Fig. 5).

### HIF1 is involved in the P4H-TM-mediated regulation of calcium entry

**Figure 8.**
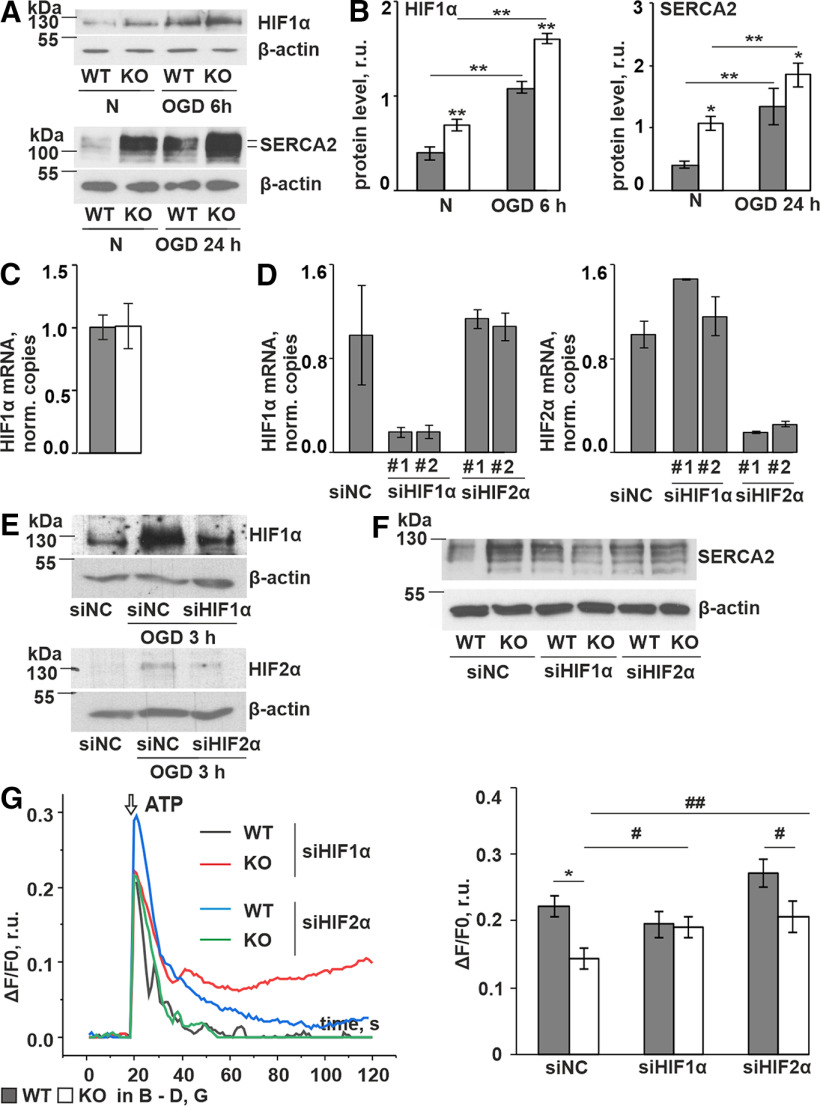
HIF1 mediates the P4H-TM effects on calcium signaling in primary cortical astrocytes. ***A***, ***B***, Western blot analysis of HIF1α stabilization (***A***, upper panel; ***B***, left panel) and SERCA2 protein expression (***A***, lower panel; ***B***, right panel) in primary cortical astrocytes incubated in normoxic (N) or oxygen (1% O_2_)-glucose deprived (OGD) conditions for 6 and 24 h, respectively. Representative blots are shown in ***A***, and the intensity of bands was quantitated by densitometry (***B***), *n* = 3 individual cultures per genotype per condition, 6 mice per genotype. β-Actin represents a loading control in (***A***). ***C***, qRT-PCR analysis of HIF1α mRNA level in normoxic astrocytes. ***D–G***, WT and P4H-TM KO astrocytes were transfected with either negative control siRNA (siNC), HIF1α siRNA (siHIF1α #1–2) or HIF2α siRNA (siHIF2α #1–2) and cultured for a further 24 h. ***D***, qRT-PCR analysis of HIF1α (left panel) and HIF2α (right panel) mRNA levels in WT astrocytes after transfection with indicated siRNAs, *n* = 2 independent cultures per siRNA. ***E***, HIF1α siRNA and HIF2α siRNA transfected WT astrocytes were exposed to OGD for 3 h and HIF1α and HIF2α stabilization was analyzed by Western blotting. Representative Western blots are shown. β-Actin represents a loading control. ***F***, Representative Western blots of SERCA2 expression in siRNA-transfected cells. β-Actin represents a loading control. ***G***, siRNA transfected astrocytes were loaded with Fluo-4 and stimulated with 100 μm ATP in a calcium-containing buffer. Representative traces of P4H-TM KO and WT astrocytes are shown on the graphs. Changes in cytosolic calcium [Ca^2+^]i peak amplitudes are shown in the diagrams. The attenuated ATP-evoked increase in [Ca^2+^]_i_ observed in P4H-TM KO cells relative to WT is eliminated by siHIF1α treatment, but not by siHIF2α, *n* = 22–33 cells per condition. Data information: data are presented as mean ± SEM in ***B–E***, ***G***; #*p* < 0.05, ##*p* < 0.01 by Student’s *t* test and *p < 0.05, **p < 0.01 by Tukey’s HSD test after one-way ANOVA method for multiple comparisons. r.u., relative unit.

As P4H-TM has been previously shown to affect HIF1 signaling (Koivunen et al., 2007; Laitala et al., 2012; Klotzsche-von Ameln et al., 2013; Leinonen et al., 2016) and as hypoxia is known to modulate calcium entry (Scott et al., 2015; Semenza and Prabhakar, 2015), we next analyzed the potential role of HIF1 and HIF2 in the P4H-TM-mediated regulation of calcium entry. In line with previous observation of HIF1α stabilization in *P4h-tm*^−/−^ cortical neurons (Leinonen et al., 2016), the amount of HIF1α was higher also in the *P4h-tm*^−/−^ astrocytes in normoxic conditions, the difference between the genotypes persisting also under OGD (Fig. 8*A*, upper panel, *B*, left panel). The increased level of HIF1α in the *P4h-tm*^−/−^ astrocytes was apparently because of stabilization of the protein, since HIF1α mRNA levels were similar in both genotypes (Fig. 8*C*). Furthermore, upregulation of the SERCA2 protein correlated with an increase in HIF1α stabilization (Fig. 8*A*, lower panel, *B*, right panel). To confirm the role of HIF1 in the SERCA2 regulation, we performed HIF1α and HIF2α siRNA knock-down experiments. The *Hif1a* and *Hif2a* expression was efficiently and specifically reduced by the siRNAs at both mRNA (Fig. 8*D*) and protein level (Fig. 8*E*). Western blotting showed that the SERCA2 protein amount in *P4h-tm*^−/−^ astrocytes was reduced to the WT levels or even lower in siHIF1α-treated cells in normoxia, while no effect was seen on siHIF2α treatment (Fig. 8*F*). Next, we assessed whether knock-down of HIF1α or HIF2α affects calcium entry in *P4h-tm*^−/−^ astrocytes by performing Fluo-4 time-lapse imaging 24 h after the transfection with siRNAs. The data show that the attenuation of ROCE in *P4h-tm*^−/−^ cells was reversed on treatment with siHIF1α, but not siHIF2α (Fig. 8*G*). Taken together, these results indicate that the higher SERCA2 expression and reduced ROCE is mediated by stabilization of HIF1 in *P4h-tm*^−/−^ astrocytes.

## Discussion

We show for the first time that P4H-TM is a major regulator of calcium signaling. Both ROCE and SOCE were affected in *P4h-tm*^−/−^ cortical astrocytes. Furthermore, calcium-dependent agonist-induced gliotransmission was downregulated in *P4h-tm*^−/−^ cells. siRNA data showed that HIF1α, but not HIF2α, is the principle downstream mediator of P4H-TM action on calcium signaling.

Several calcium sequestering ATPases, i.e., SERCA2, SERCA3, and PMCA3, were upregulated in *P4h-tm*^−/−^ astrocytes (Fig. 1*F–I*). Our results showing a decreased ATP-evoked raise of [Ca^2+^]_i_ in *P4h-tm*^−/−^ astrocytes (Fig. 2*B*) is in line with previous observations of similar calcium signaling plasticity on concomitant changes in SERCA and PMCA levels in cardiomyocytes (Ji et al., 2000), CHO cells (Brini et al., 2000), and pancreatic acinar and submandibular gland duct cells (Zhao et al., 2001).

It has been shown that PMCA and SERCA overexpression regulates the resting level of calcium in the ER of CHO cells in an opposite manner: higher [Ca^2+^]_er_ in SERCA overexpression versus lower [Ca^2+^]_er_ in PMCA overexpression (Brini et al., 2000). Our data showing a higher ionomycin-evoked increase in [Ca^2+^]_i_ in *P4h-tm*^−/−^ cells (Fig. 2*I, J*) suggests increased [Ca^2+^]_er_ resting level, pointing to a major contribution of SERCA. Based on evidence provided by the GECIs (Fig. 3), we suggest that in *P4h-tm*^−/−^ astrocytes ROCE is shaped by mitochondrial uptake, rather than by ER release and thus it is not proportional to the calcium content in the ER. Furthermore, it was previously shown that PMCAs can shape the pattern of calcium transients induced by SOCE (Pászty et al., 2015). Thus, in *P4h-tm*^−/−^ astrocytes, the enhanced increase in [Ca^2+^]_i_ on TG stimulation, and the substantial increase in [Ca^2+^]_i_ on calcium perfusion of the cells, could be the result of enhanced expression of PMCA2 and PMCA3 (Fig. 2*F, G*).

Our data on increased passive leak, measured in the presence of pharmacological modulators of leak channels (Fig. 4*A*,*B*), as well as increased expression of structural components of leak channels (Fig. 4*C–E*), suggest a compensatory mechanism to balance calcium homeostasis with increased expression of calcium sequestering ATPases in *P4h-tm*^−/−^ cells. Noteworthy, passive calcium leak via translocon is the first step of SOCE activation (Flourakis et al., 2006; Ong et al., 2007). Our results showing both enhanced passive leak and enhanced SOCE in *P4h-tm*^−/−^ astrocytes are in line with these studies.

An increase in [Ca^2+^]_i_ in astrocytes can trigger exocytotic release of gliosignals (Vardjan and Zorec, 2015). In particular, application of ATP stimulates calcium-dependent glutamate (Jeremic et al., 2001), aspartate (Duan et al., 2003), and ATP (Anderson et al., 2004; Pangrsic et al., 2007) release (Zimmermann, 2016). Calcium-dependent vesicular release of glutamate and ATP depends on the presence of the SNARE complex of proteins containing Syb2 (Zorec et al., 2012). We provide evidence using TIRF microscopy that exocytosis of Syb2-containing vesicles from astrocytes is decreased in *P4h-tm*^−/−^ cells (Fig. 5). Calcium-dependent exocytosis of gliotransmitters plays an important role in the communication between astrocytes and neurons, affects synaptic plasticity and is involved in cognitive function and several neurologic disorders (Ongür et al., 1998; Rajkowska et al., 1999; Cotter et al., 2001; Sheline et al., 2003; Banasr and Duman, 2008; Sasaki et al., 2012; Cao et al., 2013; Pannasch and Rouach, 2013). On the other hand, several steps in the vesicle release cycle are dependent on appropriate ATP levels. The supply of ATP by mitochondria is crucial for neurotransmitter release in neurons (Djeungoue-Petga and Hebert-Chatelain, 2017) and even a short interruption in ATP synthesis is sufficient to disrupt synaptic transmission in neurons (Rangaraju et al., 2014). Similarly, ATP production by mitochondria is likely to support gliotransmission in astrocytes (Jackson and Robinson, 2018). Therefore, we suggest that the observed disturbance of calcium homeostasis and modulation of intracellular ATP in *P4h-tm*^−/−^ astrocytes leads to a substantial decrease in exocytosis of gliotransmitters. This could impair the ability of *P4h-tm*^−/−^ astrocytes to modulate neuronal activity, which should be addressed in future studies. This is of particular interest regarding the involvement of P4H-TM inactivation in a severe human intellectual disability syndrome (Kaasinen et al., 2014; Rahikkala et al., 2019).

ATP synthesis during oxidative phosphorylation often correlates with O_2_ consumption, mitochondrial membrane potential (ΔΨ) and relative levels of fully assembled Complexes I, III, IV, and V (Schultz and Chan, 2001; Simonnet et al., 2014). Since none of these parameters were influenced in *P4h-tm*^−/−^ astrocytes (Fig. 6*C–F*), it is likely that the increased metabolic demands in *P4h-tm*^−/−^ astrocytes are not met by an increased production of ATP by mitochondria, and thus ultimately lead to low levels of intracellular ATP. Interestingly, although *P4h-tm*^−/−^ cells had no effect on the ΔΨ in normoxia, we observed a decrease in OGD-induced hyperpolarization (Fig. 6*D*). The mechanism of hyperpolarization is still under debate (Iijima, 2006), but it has been proposed that maintenance of ΔΨ despite respiratory inhibition is because of ATP hydrolysis by the F_1_F_O_-ATP synthase working in a reverse mode (Maldonado and Lemasters, 2014). Under ischemia, ΔΨ can be maintained as long as glycolysis provides ATP (Nieminen et al., 1994). Accordingly, dissipation of the mitochondrial membrane potential seems to be a consequence of severe energy deficit (Iijima, 2006). Thus, a decreased ability to maintain the hyperpolarized state of mitochondria during OGD can be related to the initial low cytosolic ATP content of normoxic *P4h-tm*^−/−^ astrocytes.

Enlargement of mitochondria often parallels a decrease in the numerical density of the organelles (Bertoni-Freddari et al., 1993), a phenomenon observed also in *P4h-tm*^−/−^ astrocytes (Fig. 7*B*,*C*). Previously, an inverse correlation between the size and metabolic competence of mitochondria was reported in the cerebellar cortex (Bertoni-Freddari et al., 2003) and significant mitochondrial enlargement occurs on adverse cellular conditions (e.g., oxidative stress; Bertoni-Freddari et al., 1993; Karbowski et al., 1997). It is likely that the decreased number mitochondria with enlarged size is not capable to provide adequate amounts of ATP because of its higher utilization by increased expression of calcium sequestering ATPases in *P4h-tm*^−/−^ astrocytes.

A redistribution of mitochondria was observed in *P4h-tm*^−/−^ astrocytes (Fig. 7*H*,*F*) instead of distribution throughout the arborization. Mitochondria are actively transported to sites of elevated calcium activity both in neurons and astrocytes, where they provide local energy and directly sequester calcium, thus regulating local [Ca^2+^]_i_ levels (Motori et al., 2013; Stephen et al., 2015; Rivera et al., 2016; Jackson and Robinson, 2018). The main mechanism that regulates mitochondria mobility and morphology is fusion/fission (Jackson and Robinson, 2018). The lower activity in *P4h-tm*^−/−^ astrocytes of both calcium response and vesicular exocytosis could potentially shift mitochondria mobility to prevalent fusion and thus an increase in size, accompanied with redistribution of mitochondria from the periphery toward the soma (Jackson and Robinson, 2018), but to underpin the exact molecular mechanisms responsible for the observed increase in mitochondrial size in *P4h-tm*^−/−^ astrocytes requires further studies. Activity-dependent positioning of mitochondria is crucial for synaptic transmission in neurons (Guo et al., 2005; Verstreken et al., 2005; Djeungoue-Petga and Hebert-Chatelain, 2017) and a similar role of mitochondria positioning has been proposed for gliotransmission in astrocytes (Jackson and Robinson, 2018). Therefore, the decreased density of mitochondria in distal processes of *P4h-tm*^−/−^ astrocytes may contribute to impaired gliotransmission. The lack of mitochondria in distal processes of the *P4h-tm*^−/−^ cells is especially noteworthy as emerging evidence suggests that the most important calcium transients for neuronal function occur in fine astrocyte processes, rather than in the soma (Volterra et al., 2014; Bazargani and Attwell, 2016).

Modulation of cytosolic calcium level or complete store depletion can affect HIF1α stabilization (Berchner-Pfannschmidt et al., 2004; Liu et al., 2004; Hui et al., 2006; Chai et al., 2016; Divolis et al., 2016). Constitutive stabilization of HIF1α has been shown to result in increased SERCA2 expression and diminished calcium response on T-cell receptor stimulation in thymocytes (Neumann et al., 2005). In line with our data, HIF1α was shown to mediate SERCA2b upregulation in neurons during OGD (Kopach et al., 2016). Nevertheless, although hypoxia significantly decreased the mean amplitude of caffeine-induced calcium transient in cardiomyocytes, it downregulated SERCA2 expression in these cells (Ronkainen et al., 2011; Revuelta-López et al., 2015). It is known that in cardiomyocytes, the activity of calcium pumping by SERCA2 depends on the phosphorylation status of the regulatory protein phospholamban (Haghighi et al., 2014). Thus, the different effects on SERCA2 expression by hypoxia in cardiomyocytes and P4H-TM inactivation in astrocytes, could be because of the different cell types investigated. Noteworthy, it has been shown that a combined deletion of HIF-P4H-2 and HIF-P4H-3 and hence HIF1α stabilization in cardiomyocytes leads to a drastic decrease in phospholamban expression (Xie et al., 2015). In addition, HIF1α was recently shown to directly regulate key proteins involved in SOCE, such as different isoforms of STIM and Orai, thus mediating enhanced SOCE under hypoxic conditions in several cell types (Li et al., 2015). We demonstrate here that HIF1α is a key mediator of SERCA2 overexpression as well as decreased ROCE in *P4h-tm*^−/−^ astrocytes, while HIF2α does not play a role (Fig. 8).

Taken together, our study identifies P4H-TM as a novel regulator of several aspects of calcium signaling in astrocytes. In addition, we show that HIF1α is the key mediator between P4H-TM and calcium signaling. Besides the microarray analysis of the whole cortex and qRT-PCR analyses of selected key genes in the astrocytes reported in this study, transcriptome analysis of the WT and *P4h-tm*^−/−^ astrocytes either by microarray analysis or RNA-seq would be of interest in the future to reveal possible further effects of P4H-TM on genes involved in calcium signaling. In addition, whether inactivation of P4H-TM has similar effects on calcium signaling in other cell types remains to be studied. Furthermore, future studies are required to analyze what effects P4H-TM has on astrocyte functions in for example various disease settings, and to what extent they are caused by the effects on calcium signaling observed in this study. For example, it will be of interest to determine whether the observed effects of P4H-TM on calcium signaling and vesicular transport will provide novel information on the etiology of the human HIDEA disease caused by P4H-TM mutations (Kaasinen et al., 2014; Rahikkala et al., 2019) and the behavioral phenotype of increased social behavior, decreased anxiety, and absence of despair of the *P4h-tm*^−/−^ mice (Leinonen et al., 2019).

## Synthesis

Reviewing Editor: Deanna Smith, University of South Carolina Decisions are customarily a result of the Reviewing Editor and the peer reviewers coming together and discussing their recommendations until a consensus is reached. When revisions are invited, a fact-based synthesis statement explaining their decision and outlining what is needed to prepare a revision will be listed below. The following reviewer(s) agreed to reveal their identity: James Goldman. Note: If this manuscript was transferred from JNeurosci and a decision was made to accept the manuscript without peer review, a brief statement to this effect will instead be what is listed below.

Synthesis

Both reviewers agreed that the study documents a wide range of phenotypes in P4h-TM mutant astrocytes. However, the reviewers differed with respect to the need for addition experiments. Reviewer 1 had no concerns initially, while reviewer two had several concerns related to mechanism. After discussion, we concluded that while analysis of altered astrocyte function or transcriptome analysis would increase the impact of the study, either would require more than two months and would be difficult given the complexity of the system. The authors should clearly address these limitations of the study in the discussion and should indicate how future experimentation could fill in gaps. Also, both reviewers agreed that the specific comments from reviewer 2 should be addressed in the revised manuscript. Reviewer 2 had suggestions for how to address comments 8 and 9. These are included in italics after the comments themselves.

Reviewer # 1:

The study describes phenotypes in cortical gene expression and calcium homeostasis of cultured astrocytes from prolyl 4-hydrolase (P4h) knock-out mice. However, the molecular mechanisms underlying these phenotypes were not investigated. Accordingly, the impact of the study is limited.

I have no concerns.

Reviewer # 2

The study provides a modest advance to understanding the regulation of calcium signaling by prolyl-hydrolases in astrocytes. The consequences of the changes in calcium signaling to the function of astrocytes is not clear.

Byts et al. have investigated how P4H-TM prolyl 4-hydrolase may regulate calcium signaling and calcium transients by studying cultured mouse astrocytes derived from a P4H knockout (KO) line. By comparing astrocytes from wild type mice to KO mice, they provide evidence that PH4-TM is important for calcium signaling, including both receptor-mediated and store-operated calcium release and entry, and that HIF1 may be a further mediator of the effects of PH4-TM. The study does partially reveal underlying mechanisms but some of the conclusions are based only on indirect measures. The authors do not examine how the changes in calcium signaling produced in the KO astrocytes changes astrocyte function.

Furthermore, the microarray analysis uses whole cortex (I assume cortex and underlying white matter together) and finds changes in a number of genes that are involved in the regulation of calcium signaling. This gives an average of changes in all cell types. The authors have then chosen to examine isolated mouse astrocytes, arguing that astrocytes have important secretory roles in the brain, although they do not directly show a loss of secretory activity. The findings would have more impact if the authors were to find similar transcriptional changes in astrocytes isolated from the KO mouse brains.

Specific comments:

1. What does “enrichment for astroglial cells was about 90%” mean? What other cell types were present in the cultures?

2. The KO astrocytes showed an upregulation of PMCA2 and 3 and SERCA3 genes and increased SERCA2 and PMCA3 proteins. Did the authors try Western blots with antibodies to PMCA2?

3. It might be useful to the reader to describe briefly the Hif-p4h-2 hypomorph mutation and gene expression.

4. It is still not clear why the calcium content in ER is higher in the KO cells. Is it because levels of ATPases are increased? Transcriptome assays show upregulation, but were the levels of ATPase proteins also increased?

5. Anisomycin treatment would inhibit translocation and thus levels of many proteins, particularly those with rapid turnover rates. The authors should discuss this issue.

6. The higher phosphorylation levels of elF2, Erk1/2 and p38 kinases are not specific for the ER stress response. The authors could look for more specific responses.

7. The authors should explain how the ER stress response fits into their model Why do these changes take 6 hours to increase significantly?

8. TIRF shows a decrease in fusion events in the KO astrocytes. They are measuring the appearance of reporter at the cell surface. A decrease in the numbers of reporter puncta could mean either that there is a decrease in fusion and/or that there is an increase in the disappearance of the reporter from the cell surface, through endocytosis or other means.

Comment from Reviewer 1 related to 8: Since the study uses pH-sensitive Syb2-pHluorin it is very justified to conclude that the appearance of new punctae in response to stimulation represents exocytotic events. Since resting levels are also reduced in the mutants, the evoked counts should be normalized to the resting levels to better quantify levels of exocytosis. In addition, the authors should ensure that Syb2-pHluorin as well as endogenous Syb levels are similar in control and mutant cells. The interpretation of the decreased resting levels is much more difficult since the reduced number of punctae at rest could be caused by reduced exocytosis or increased endocytosis. Experimentally differentiating between these possibilities is not necessary in view of the evoked data but the data at rest should be properly explained the MS.

9. The decrease in fusion events could imply a loss of secretory functions. However, this is only an indirect measure of secretion. The study would be stronger if the author were to measure the secretion of specific molecules and show a decrease in secretion.

Comment from Reviewer 1 related to 8: Direct secretion data are not superior to the Syb2-pHluorin data since they depend on an even larger multitude of parameters including reuptake, which is difficult to control. Therefore, I suggest to only solidify the Syb2-pHluorin data.

10. Are levels of Syb2 changed in the KO cells?

11. What might an increased size of mitochondria imply? Increased mitochondrial fusion?

12. Figure 7A KO shows two vesicles, which look like coated vesicles produced by endocytosis.

Were only coated vesicles counted in the quantitation?

13. Figure 7H requires quantitation. Only a few “representative” astrocytes are shown.

14. Did Western blotting after HIF1,2a siRNA treatment show loss of these proteins?

15. Is the increased level of HIF1a in the KO astrocytes due to stabilization of the protein?

## Author Response

Subject: Revision of eNeuro eN-TNWR-0253-20X

Dear Dr. Smith,

We were very pleased to learn that our manuscript was judged potentially suitable for publication in eNeuro, provided appropriate revisions are made. We are also extremely thankful for the 2-month extension granted for revision due to special circumstances imposed by certain Covid-19 restrictions. We have now revised the manuscript according to the recommendations and hope that they adequately address the reviewers’ concerns. Below, you can find our point-by-point reply to the reviews and the revisions are indicated in a colored font in the revised manuscript. We sincerely thank you and the reviewers for your valuable comments and hope that you will find the revised manuscript suitable for publication in eNeuro.

Yours sincerely,

The corresponding author

---------------------------------------------

Synthesis of Reviews:

Computational Neuroscience Model Code Accessibility Comments for Author (Required):

N/A

Significance Statement Comments for Author (Required):

Include a sentence about the types of future studies that could strengthen the hypothesis.

Response: The following sentence has been added to the end of the Significance Statement: “Therefore, future studies to unravel the exact molecular mechanisms how P4H-TM affects calcium signaling and what effects P4H-TM has on astrocyte functions in healthy and various disease conditions will be of interest.” To accommodate the word limit the following parts of the original text of the significance statement were removed (indicated with strikethrough): “P4H-TM is a recently identified member of the prolyl 4-hydroxylase family. Biallelic loss of function P4H-TM mutations have been shown to cause a syndromic form of severe cognitive impairment in humans. However, the cellular level effects of P4H-TM deficiency have not been studied yet. Here we demonstrate for the first time that inactivation of P4H-TM in astrocytes disturbs calcium signaling in a HIF1 dependent manner. The observed changes in calcium signaling in the absence of P4H-TM activity were accompanied by attenuated vesicular exocytosis. These findings suggest that abnormal calcium signaling resulting from P4H-TM inactivation may be involved in the molecular basis of a severe human intellectual disability syndrome.”

Comments on the Visual Abstract for Author (Required):

N/A

Synthesis Statement for Author (Required):

Synthesis

Both reviewers agreed that the study documents a wide range of phenotypes in P4h-TM mutant astrocytes. However, the reviewers differed with respect to the need for addition experiments. Reviewer 1 had no concerns initially, while reviewer 2 had several concerns related to mechanism. After discussion, we concluded that while analysis of altered astrocyte function or transcriptome analysis would increase the impact of the study, either would require more than two months and would be difficult given the complexity of the system. The authors should clearly address these limitations of the study in the discussion and should indicate how future experimentation could fill in gaps. Also, both reviewers agreed that the specific comments from reviewer 2 should be addressed in the revised manuscript. Reviewer 2 had suggestions for how to address comments 8 and 9. These are included in italics after the comments themselves.

Response: We sincerely thank the reviewers and editor for the conclusion. We acknowledge and fully understand that we have not yet solved entirely the mechanisms how P4H-TM regulates calcium signaling and what effects that has on astrocyte functions. As stated in our previous response to reviews from the Journal of Neuroscience that were included in the transfer to eNeuro, very little is currently known of the cellular functions of P4H-TM, a relatively unexplored member of the prolyl 4-hydroxylase family, other members of which are known to have prominent roles in the regulation of collagen synthesis and hypoxia response. P4H-TM mutations are known to cause a very severe intellectual disability syndrome in humans and we therefore strongly believe that the current manuscript provides important first data on cellular effects of the lack of P4H-TM. Regarding the exact P4H-TM mechanisms, at least part of them are mediated by enhanced stabilization of HIF upon inactivation of P4H-TM (as shown in the last results section of the current manuscript). However, due to the high likelihood that P4H-TM has novel yet undiscovered substrates, other mechanisms may be involved. We have been and are carrying out extensive substrate screening studies with recombinant P4H-TM, but unfortunately with no definite hits yet. Even with our extensive and I would say internationally unique capabilities in producing recombinant P4Hs as active enzymes, P4H-TM has turned out to be a very tricky one in this respect, probably due to its transmembrane nature. I strongly believe that in the meanwhile the current manuscript provides data that gives highly important background for the ultimate aim of identifying the exact P4H-TM mediated mechanisms. We now address limitations of the current study in the discussion (p. 34) and in appropriate places in the results and discussion as indicated below. In addition, we now more clearly state in the introduction what is known of the role of P4H-TM in the regulation of HIF and why P4H-TM is likely to have also other substrates (p. 5-6).

Reviewer # 1:

The study describes phenotypes in cortical gene expression and calcium homeostasis of cultured astrocytes from prolyl 4-hydrolase (P4h) knock-out mice. However, the molecular mechanisms underlying these phenotypes were not investigated. Accordingly, the impact of the study is limited.

I have no concerns.

Response: Although we fully understand and appreciate the reviewer’s opinion, we would like to refer to our above response to the synthesis statement.

Reviewer # 2

The study provides a modest advance to understanding the regulation of calcium signaling by prolyl-hydrolases in astrocytes. The consequences of the changes in calcium signaling to the function of astrocytes is not clear.

Byts et al. have investigated how P4H-TM prolyl 4-hydrolase may regulate calcium signaling and calcium transients by studying cultured mouse astrocytes derived from a P4H knockout (KO) line. By comparing astrocytes from wild type mice to KO mice, they provide evidence that PH4-TM is important for calcium signaling, including both receptor-mediated and store-operated calcium release and entry, and that HIF1 may be a further mediator of the effects of PH4-TM. The study does partially reveal underlying mechanisms but some of the conclusions are based only on indirect measures. The authors do not examine how the changes in calcium signaling produced in the KO astrocytes changes astrocyte function.

Furthermore, the microarray analysis uses whole cortex (I assume cortex and underlying white matter together) and finds changes in a number of genes that are involved in the regulation of calcium signaling. This gives an average of changes in all cell types. The authors have then chosen to examine isolated mouse astrocytes, arguing that astrocytes have important secretory roles in the brain, although they do not directly show a loss of secretory activity. The findings would have more impact if the authors were to find similar transcriptional changes in astrocytes isolated from the KO mouse brains.

Response: To our understanding very little, if anything, is currently known about the individual roles of prolyl hydroxylases in the regulation of calcium signaling in astrocytes. In Fig. 2 we provide novel data that clearly shows that the outcome of P4H-TM inactivation on calcium entry is different from those of HIF-P4Hs 1-3. We fully understand the critique that consequences of the changes in calcium signaling to the function of astrocytes is not clear and highly appreciate the synthesis conclusion of the reviewers and the editor that while analysis of altered astrocyte function would increase the impact of the study, it would require a long time and would be difficult given the complexity of the system. This will be very interesting to address in future studies and we acknowledge this limitation in the revised manuscript (p. 34). Regarding transcription analysis we agree that it would be interesting to carry out a transcriptome analysis of the isolated astrocytes also and acknowledge this limitation in the revised manuscript (p. 34). However, we would like to emphasize that we carried out qRT-PCR analysis of the expression of the major genes of interest to this study in primary astrocytes isolated from the KO mouse brains (Figs. 1F, 2G, 3F, 4C). Therefore, we highly appreciate the synthesis statement concluding that while analysis of astrocyte transcriptome would increase the impact of the study, it would require a long time and would be difficult given the complexity of the system.

Specific comments:

1. What does “enrichment for astroglial cells was about 90%” mean? What other cell types were present in the cultures?

Response: Most protocols for preparing astroglial-enriched cultures from rat/mouse late embryos/neonates are derived from the seminal work of Booher and Sensenbrenner or the later modification by McCarthy and de Vellis (which we have used here). In these protocols the dissected cortex is dissociated by mechanical and/or enzymatic digestion and the dissociated cells are plated. Astrocytes proliferate rapidly and a confluent culture is obtained generally 7-14 days after plating. Since astrocytes constitute the most abundant cell type in these preparations, the cultures are described as astroglial-rich or astroglial-enriched. However, in none of these preparations astrocytes represent 100% of the cells (Saura J. Microglial cells in astroglial cultures: a cautionary note Journal of Neuroinflammation 2007, 4:26). Depending on culture conditions, oligodendrocytes, neurons, various types of precursors, ependymal cells, fibroblasts, endothelial cells or microglial cells can be present in these cultures, generally in small proportions. We now determined the content of microglial cells and neurons in our preparations (see representative pictures below). The microglia content was about 4% and neuronal content about 2% as detected by immunocytochemistry for CD11b and β-tubulin III cell type markers, respectively. GFAP was used to identify astrocytes. This information is now given in p. 9 of the revised manuscript.

We also noticed that we had accidentally omitted the vendor information of certain chemicals used in the study. We apologize for this and the information is now added to p. 9 of the revised manuscript.

GFAP, DAPI CD11b, DAPI beta-tubulin III, DAPI

2. The KO astrocytes showed an upregulation of PMCA2 and 3 and SERCA3 genes and increased SERCA2 and PMCA3 proteins. Did the authors try Western blots with antibodies to PMCA2?

Response: We have now carried out Western blot analysis with a PMCA2 specific antibody. However, there was no difference in the protein level of PMCA2 between the P4H-TM WT and KO mice (p. 18 and new Fig. 1G in the revised manuscript). Therefore, the observed ∼1.5-fold induction in the PMCA2 mRNA expression level was not sufficient to be manifested at protein level.

3. It might be useful to the reader to describe briefly the Hif-p4h-2 hypomorph mutation and gene expression.

Response: We have added a short description of the Hif-p4h-2 hypomorph mutation and gene expression into the revised manuscript (p. 7).

4. It is still not clear why the calcium content in ER is higher in the KO cells. Is it because levels of ATPases are increased? Transcriptome assays show upregulation, but were the levels of ATPase proteins also increased?

Response: Yes, as shown in the original manuscript (Fig. 1F in the original manuscript, Fig. 1H in the revised manuscript, ATP2A2/SERCA2 is significantly upregulated at protein level in P4H-TM KO astrocytes. Unfortunately (as stated in the original and revised manuscript), variable and nonreproducible results depending on the antibody source were obtained for SERCA3 expression and thus conclusions of SERCA3 protein expression level could not be made despite the significant upregulation of SERCA3 mRNA level. As it has been shown that overexpression of ER-located calcium ATPases regulates the resting level of calcium in the ER (Brini M, Bano D, Manni S, Rizzuto R, & Carafoli E (2000) Effects of PMCA and SERCA pump overexpression on the kinetics of cell Ca2+ signalling. EMBO J 19:4926-4935), we suggest in the discussion of the original and revised manuscript (p. 29-30) that the observed SERCA upregulation in P4H-TM KO cells can be the cause of higher ER calcium content.

5. Anisomycin treatment would inhibit translocation and thus levels of many proteins, particularly those with rapid turnover rates. The authors should discuss this issue.

Response: In our experiments anisomycin was added 10 min prior to thapsigargin to inhibit calcium permeability of the translocon. The 10-min anisomycin pretreatment is too short for marked changes in protein levels due to translation inhibition (Claydon AJ, Beynon R (2012) Proteome dynamics: revisiting turnover with a global perspective. Mol Cell Proteomics 11:1551-65). Typically, about 2-h inhibition by anisomycin is needed to detect significant reductions in protein levels (Dynamic visualization of local protein synthesis in hippocampal neurons. Aakalu G, Smith WB, Nguyen N, Jiang C, Schuman EM. Neuron. 2001 May;30(2):489-502.). This issue and considerations are now discussed in p. 23.

6. The higher phosphorylation levels of elF2, Erk1/2 and p38 kinases are not specific for the ER stress response. The authors could look for more specific responses.

Response: TG-induced translocon-associated calcium loss is known to contribute to ER stress response modulation and the resulting Erk-elf2α overactivation in astrocytes has been shown to be associated with a distinct pathogenic reactivity state and decreased secretome (Kamiya T, Obara A, Hara H, Inagaki N, & Adachi T (2011) ER stress inducer, thapsigargin, decreases extracellular-superoxide dismutase through MEK/ERK signalling cascades in COS7 cells. Free Radic Res 45:692-698; Johnson GG, White MC, Wu JH, Vallejo M, & Grimaldi M (2014) The deadly connection between endoplasmic reticulum, Ca2+, protein synthesis, and the endoplasmic reticulum stress response in malignant glioma cells. Neuro Oncol 16:1086-1099; Martin-Jimenez CA, Garcia-Vega A, Cabezas R, Aliev G, Echeverria V, Gonzalez J, & Barreto GE (2017) Astrocytes and endoplasmic reticulum stress: A bridge between obesity and neurodegenerative diseases. Prog Neurobiol 158:45-68; Smith HL, Freeman OJ, Butcher AJ, Holmqvist S, Humoud I, Schätzl T, Hughes DT, Verity NC, Swinden DP, Hayes J, de Weerd L, Rowitch DH, Franklin RJM & Mallucci GR (2020) Astrocyte unfolded protein response induces a specific reactivity state that causes non-cell-autonomous neuronal degeneration. Neuron. 105:855-866). Furthermore, the p38 MAPK pathway is known to respond to various cellular and extracellular stresses and is activated by TG treatment (Kim DS, Kim JH, Lee GH, Kim HT, Lim JM, Chae SW, Chae HJ & Kim HR (2010) p38 Mitogen-activated protein kinase is involved in endoplasmic reticulum stress-induced cell death and autophagy in human gingival fibroblasts. Biol Pharm Bull. 33:545-9; Huang Y, Li X, Wang Y, Wang H, Huang C, & Li J (2014) Endoplasmic reticulum stress-induced hepatic stellate cell apoptosis through calcium-mediated JNK/P38 MAPK and Calpain/Caspase-12 pathways. Mol Cell Biochem 394:1-12; Darling NJ & Cook SJ (2014) The role of MAPK signalling pathways in the response to endoplasmic reticulum stress. Biochim Biophys Acta. 1843:2150-63. Therefore, we were particularly interested in analyzing the phosphorylation levels of these proteins. We completely agree that there are more specific ER markers and have revised this part of the manuscript to results text to better indicate the motivation behind the phosphorylation analysis and conclude that the observed changes in temporal kinase phosphorylation patterns upon TG treatment in P4H-TM KO astrocytes relative to WT could indicate enhanced susceptibility to ER stress and detailed analyses of the effects of P4H-TM inactivation on the induction of ER stress with various stress inducers, markers and outcomes should be addressed in future studies (p. 23-24).

7. The authors should explain how the ER stress response fits into their model. Why do these changes take 6 hours to increase significantly?

Response: The TG-induced phosphorylation of Erk and elF2α has a temporal pattern, the initial increase in phosphorylation plateauing typically at around 2 h followed by a progressive decrease (Smith et al., 2020). As seen in Fig. 4F, G the increase in phosphorylation upon TG treatment occurs quickly and is observed already at 10 min and the change in the phosphorylation between the genotypes is in its persistence. As described in the response above we have rewritten this part of the results to make it clearer (p. 23-24).

8. TIRF shows a decrease in fusion events in the KO astrocytes. They are measuring the appearance of reporter at the cell surface. A decrease in the numbers of reporter puncta could mean either that there is a decrease in fusion and/or that there is an increase in the disappearance of the reporter from the cell surface, through endocytosis or other means.

Comment from Reviewer 1 related to 8: Since the study uses pH-sensitive Syb2-pHluorin it is very justified to conclude that the appearance of new punctae in response to stimulation represents exocytotic events. Since resting levels are also reduced in the mutants, the evoked counts should be normalized to the resting levels to better quantify levels of exocytosis. In addition, the authors should ensure that Syb2-pHluorin as well as endogenous Syb levels are similar in control and mutant cells. The interpretation of the decreased resting levels is much more difficult since the reduced number of punctae at rest could be caused by reduced exocytosis or increased endocytosis. Experimentally differentiating between these possibilities is not necessary in view of the evoked data but the data at rest should be properly explained the MS.

Response: We agree with the reviewer that the interpretation of the TIRF data at rest before the ATP treatment is difficult, since the disappearance of the reporter from cell surface can occur both through exocytosis and endocytosis. Nevertheless, it is a well justified and an often-used way to measure exocytosis in case of a signal evoked by ATP treatment (as pinpointed by reviewer#1). Now we clearly state this in the results part (p. 25). We have also solidified our TIRF data as suggested by reviewer#1 by normalizing the evoked counts by signal at rest (p. 13, Fig. 5A and B, right panels). After normalization the difference in the evoked signal between the WT and KO cells was even more prominent and significant. As an additional control as suggested by reviewer#1 we also confirmed by Western blotting that Syb2-pHluorin and endogenous Syb2 protein levels do not differ between the WT and KO cells (p. 25 and new Fig. 5 D, E).

9. The decrease in fusion events could imply a loss of secretory functions. However, this is only an indirect measure of secretion. The study would be stronger if the author were to measure the secretion of specific molecules and show a decrease in secretion.

Comment from Reviewer 1 related to 9: Direct secretion data are not superior to the Syb2-pHluorin data since they depend on an even larger multitude of parameters including reuptake, which is difficult to control. Therefore, I suggest to only solidify the Syb2-pHluorin data.

Response: We agree with Reviewer#1 and have solidified the Syb2-pHluorin data (Fig.5A and B, right panels; Fig. 5 D, E) as described in the above response.

10. Are levels of Syb2 changed in the KO cells?

Response: We have now analyzed the protein levels of both endogenous Syb2 (in untransfected cells) and Syb2-pHluorin (in transfected cells) by WB using anti-Syb2 and anti-GFP antibodies. The levels were equal between genotypes (new Fig. 5D, E).

11. What might an increased size of mitochondria imply? Increased mitochondrial fusion?

Response: Indeed, the main mechanism that regulates mitochondria mobility and morphology is fusion/fission. The increased size of mitochondria in the KO cells could very well be due to increased fusion, and we discuss this now in the revised manuscript, p. 32: “The main mechanism that regulates mitochondria mobility and morphology is fusion/fission (Jackson & Robinson, 2018). The lower activity in P4h-tm-/- astrocytes of both calcium response and vesicular exocytosis could potentially shift mitochondria mobility to prevalent fusion and thus an increase in size, accompanied with redistribution of mitochondria from the periphery towards the soma (Jackson & Robinson, 2018), but to underpin the exact molecular mechanisms responsible for the observed increase in mitochondrial size in P4h-tm-/- astrocytes requires further studies.” Jackson JG & Robinson MB (2018): Regulation of mitochondrial dynamics in astrocytes: Mechanisms, consequences, and unknowns. Glia 66(6):1213-1234.

the existence of a population of diminutive mitochondria

(0.2 mm in diameter) in the very distal tips of the peripheral astrocyte

processes was described in astrocyte cultures

12. Figure 7A KO shows two vesicles, which look like coated vesicles produced by endocytosis.

Were only coated vesicles counted in the quantitation?

Response: We have counted both coated and uncoated vesicles and this is now stated in p. 16.

13. Figure 7H requires quantitation. Only a few “representative” astrocytes are shown.

Response: We have quantified the number of cells with clear punctate pattern of mitochondria staining in the distal processes (phenotype 1) versus the number of cells with distal processes virtually devoid of any mitochondrial staining (phenotype 2) (p. 17, new Fig. 7I). In contrast to WT, the KO cell cultures were enriched in phenotype 2.

14. Did Western blotting after HIF1,2a siRNA treatment show loss of these proteins?

Response: We confirmed that HIF1,2 siRNA treatment shows loss of these proteins by Western blotting (p. 28, new Fig. 8E). Because HIF1 and HIF2 proteins/α subunits are present only in hypoxic cells we analyzed this under oxygen glucose deprivation.

15. Is the increased level of HIF1a in the KO astrocytes due to stabilization of the protein?

Response: As described in the introduction of the manuscript, the major mechanism that regulates HIF1α protein level is oxygen/prolyl hydroxylase dependent regulation of HIF1α stability and we have also shown previously that P4H-TM is capable for this (Koivunen et al, 2007). Furthermore, we analyzed the HIF1α mRNA level and now show that there is no upregulation of HIF1α mRNA level in P4H-TM KO astrocytes (p. 28, new Fig. 8C).
